# AuBr_3_-catalyzed azidation of per-*O*-acetylated and per-*O*-benzoylated sugars

**DOI:** 10.3762/bjoc.14.56

**Published:** 2018-03-22

**Authors:** Jayashree Rajput, Srinivas Hotha, Madhuri Vangala

**Affiliations:** 1 Department of Chemistry, Indian Institute of Science Education and Research, Pune 411 008, India

**Keywords:** acylated sugars, azidation, gold(III) bromide, *N*-glycoside, oxophilicity

## Abstract

Herein we report, for the first time, the successful anomeric azidation of per-*O*-acetylated and per-*O*-benzoylated sugars by catalytic amounts of oxophilic AuBr_3_ in good to excellent yields. The method is applicable to a wide range of easily accessible per-*O*-acetylated and per-*O*-benzoylated sugars. While reaction with per-*O*-acetylated and per-*O*-benzoylated monosaccharides was complete within 1–3 h at room temperature, the per-*O*-benzoylated disaccharides needed 2–3 h of heating at 55 °C.

## Introduction

The past few decades had seen the enrichment of transition metal complexes in various glycosylation strategies [1]. In particular, gold complexes with their operationally simple, safe and neutral reaction conditions, had widely contributed to the development of new glycosylation methods. Gold(I) and gold(III) complexes are usually alkynophilic [2], carbophilic and oxophilic because of their affinity towards the alkynes’ and C–O π systems [3–6]. Thus, various research groups employed either a remote alkyne group possessing versatile glycosyl donors [7–16] or used glycals [17] for effective *O*-, *C*-, and *S*-glycosylation reactions using gold(I) and gold(III) catalysts. Among the gold-catalyzed activation of non-alkynic glycosyl donors, glycosyl halides [18], armed *O*-methyl glycosides [19], armed and disarmed thioglycosides [20] as well as trichloroacetimidate [21–22] donors were successfully applied to *O*- and *C*-glycosylations.

Of the gold-catalyzed *N*-glycosylation reactions, Yu et al. demonstrated the effective purine and pyrimidine nucleoside synthesis using per-*O*-acyl/per-*O*-benzoyl furanosyl and pyranosyl *o*-hexynylbenzoates [23]. Subsequently, Hotha and co-workers utilized propargyl 1,2-orthoesters and alkynyl glycosyl carbonate donors for the synthesis of pyrimidine nucleosides [24–25]. In addition, *N*-glycosides are also accessible by AuCl_3_/phenylacetylene-promoted Ferrier rearrangement of glycals [17], thus, demonstrating the efficient catalysis by alkynophilic and carbophilic Au complexes. Although the alkynophilicity and carbophilicity of Au complexes are well explored, very little is known about the role played by the oxophilicity of gold [26] towards the glycosylation reactions.

Generally, easily accessible per-*O*-acetylated and per-*O*-benzoylated sugars are not regarded as effective glycosyl donors in glycosylation reactions since they require harsh reaction conditions due to the deactivating effect of the ester groups. In a recently reported gold(III)-mediated reaction Vankar and co-workers disclosed that a AuCl_3_–phenylacetylene complex promotes the *O*-glycosylation of armed 1-*O*-acetyl pyranosides and furanosides [17,27]. The authors also observed that 5 mol % AuCl_3_ alone promoted the *O*-glycosylation albeit in low yields, thus indicating the possible utility of the oxophilic character of Au(III) towards the acetylated sugars.

Among the *N*-glycosides, anomeric azido glycosides are important intermediates due to various applications in the synthesis of various glycosyl amides [28–29], glycoconjugates [30–32], *N*-glycosyl heterocycles [33–34], *N*-glycosyl triazole [35–36], etc. Glycosyl azides can be accessed from the corresponding glycosyl halides [37–40] by nucleophilic displacement with NaN_3_ or using trimethylsilyl azide in the presence of a phase transfer catalyst [41–45]. More commonly, glycosyl azides are synthesized from per-*O*-acetylated sugars using trimethylsilyl azide in the presence of a variety of Lewis acids such as SnCl_4_ [46], TiCl_4_ [47–48], BF_3_·OEt_2_ [49]_,_ TMSOTf [50–51], etc. However, at higher concentration Lewis acids can potentially lead to slow anomerization [52]. In 2011, Chen’s group reported that 5 mol % FeCl_3_ can catalyze the reaction of trimethylsilyl azide with per-*O*-acetylated β-monosaccharides to afford glycosyl azides in 3–7 h, whereas per-*O*-acetylated β-di- and trisaccharides required 22–28 h for complete conversion [53]. Despite the use of various Lewis acid catalysts, gold(III)-catalyzed azidation reactions remain rather underexplored till date. In our efforts towards the syntheses of glycoderivatives [54–56], we found that AuBr_3_ activates per-*O*-acetylated and per-*O*-benzoylated sugars towards anomeric azidation in good to excellent yields.

## Results and Discussion

**Scheme 1:**
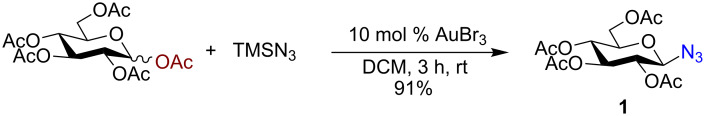
Azidation of per *O*-acetylated glucose.

We began our studies by treating per-*O*-acetylated glucose with 3 equiv trimethylsilyl azide in the presence of 10 mol % AuBr_3_ in dichloromethane at room temperature. The reaction proceeded smoothly giving 2,3,4,6-tetra-*O*-acetyl-β-D-glucopyranosyl azide (**1**) within 3 h in 91% yield (Scheme 1).

Next we tested the slightly more Lewis acidic AuCl_3_ in this reaction, and found that 10 mol % of AuCl_3_ were essential for complete consumption of the starting material. As AuBr_3_ is less hygroscopic than AuCl_3_ and thus easier to handle, all further experiments were conducted with AuBr_3_ only. Interestingly, we noticed that, when stirring the reaction mixture with 4 Å molecular sieves powder to remove moisture prior to the addition of the catalyst no product was formed. This observation suggested that in addition to the coordination of AuBr_3_ to the lone pairs of the anomeric acetate carbonyl oxygen, probably the Brønsted acid, HBr, generated from AuBr_3_ and water present in the reaction medium is also participating in the catalytic cycle.

Also no reaction was observed when peracetylated galactose and 3 equiv of trimethylsilyl azide were stirred at room temperature in the absence of AuBr_3_ as the catalyst. Additionally, the treatment of peracetylated galactose with NaN_3_ instead of trimethylsilyl azide at room temperature for 6 h also yielded no product. In view of the above observations, a plausible catalytic cycle is proposed in Supporting Information File 1.

In a similar fashion using 10 mol % AuBr_3_, 2,3,4,6-tetra-*O*-acetyl-β-D-galactopyranosyl azide (**2**) and 2,3,4,6-tetra-*O*-acetyl-β-D-mannopyranosyl azide (**3**) were obtained from their corresponding per-*O*-acetylated sugar precursors (Table 1, entries 1 and 2) in 3 h at 25 °C in 87% and 90% yield, respectively. Interestingly, the reaction of per-*O*-acetylated xylopyranose (Table 1, entry 3) also proceeded smoothly affording 2,3,4-tri-*O*-acetyl-β-D-xylopyranosyl azide in 85% yield after 1 h. Conversely, the azidation of peracetylated L-fucopyranose gave an α/β mixture (1:6), with 2,3,4-tri-*O*-acetyl-β-L-fucopyranosyl azide (**5**) obtained in 71% yield in 1 h [57]. Gratifyingly, the disaccharide, β-D-cellobiosyl azide (**6**), could be conveniently synthesized from the commercially available α-D-peracetylated cellobiose in 3 h at room temperature in an excellent yield. Additionally, peracetylated maltotriose took 5 h for completion to afford the corresponding azido compound **7** in 82% yield.

**Table 1:** Scope of AuBr_3_-catalyzed azido glycosylation of peracetates.

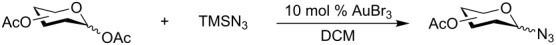				

Entry^a^	Substrate	Product	Time (h)/ temp	α:β ratio/ yield^b^

1	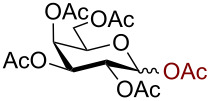	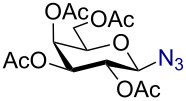 **2**	3/rt	α:β 1:19 β-87%
2	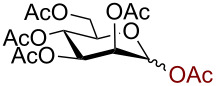	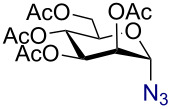 **3**	3/rt	α:β 19:1 α-90%
3	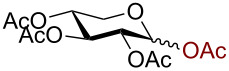	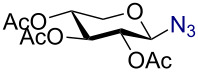 **4**	1/rt	β-only β-85%
4	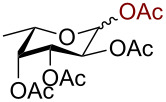	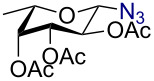 **5**	1/rt	α:β 1:6 β-71%
5	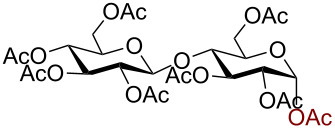	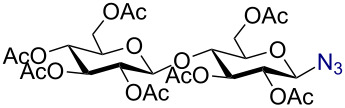 **6**	3/rt	β-only β-92%
6^c^	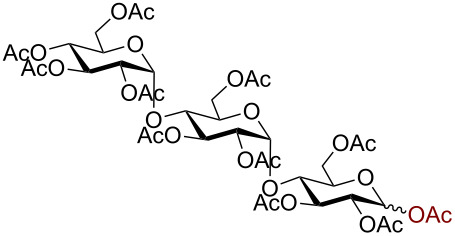	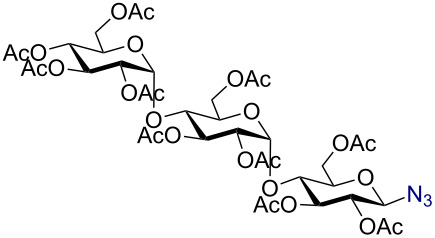 **7**	5/rt	β-only β-82%
7	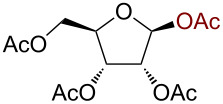	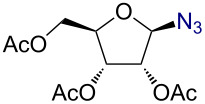 **8**	2/0 °C	β-only β-93%
8^d^	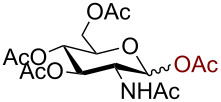	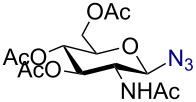 **9**	48/55 °C	β-only β-74%

^a^All reactions were carried out on a 300 mg scale, using 10 mol % AuBr_3_ and 3 equiv TMSN_3_ in 4 mL of CH_2_Cl_2_; ^b^isolated purified yield; ^c^30 mol % AuBr_3_ were used; ^d^1 equiv AuBr_3_ was used; rt: room temperature.

As anticipated, the anomeric azidation of peracetylated ribofuranose (Table 1, entry 7) proceeded well even at 0 °C within 2 h to give the product in 93% yield. However, the azidation of peracetylated 2-deoxy-D-glucosamine was slow and required one equivalent of AuBr_3_ and heating at 55 °C for 48 h to reach completion. In this case the desired product β-azido 2,3,4,6-acetyl-D-glucosamine (**9**) could be obtained in 74% yield. The need of using higher amounts of catalyst in this reaction could be attributed to the possible coordination of AuBr_3_ with the amide.

Having successfully accomplished the gold(III)-catalyzed azido glycosidation of per-*O*-acetates, we next turned our attention to per-*O*-benzoylated sugars. Gratifyingly, using 12 mol % AuBr_3_, the easily accessible per-*O*-benzoylated mannopyranose and glucopyranose (Table 2, entries 1 and 2) were readily converted into the corresponding 2,3,4,6-tetra-*O*-benzoyl-α-D-mannopyranosyl azide (**10**) and 2,3,4,6-tetra-*O*-benzoyl-β-D-glucopyranosyl azide (**11**) in excellent yields within 3 h reaction at room temperature. It is noteworthy that the present method can be successfully applied to perbenzoylated sugars with a slightly higher catalyst loading given the fact that they these sugars are more deactivated than the corresponding acetates. Conversely, the reaction of 1,2,3,4-tetra-*O*-benzoyl-L-rhamnopyranoside (Table 2, entry 3) proceeded within 1 h at room temperature giving 2,3,4-tri-*O*-benzoyl-α-L-rhamnopyranosyl azide (**12**) in 71% yield. Furthermore, C5-*O*-TBDPS-protected perbenzoylated arabinofuranose (Table 2, entry 4) afforded the desired azide **13** in 70% yield along with some amounts of desilylated product. Furthermore, azidation of perbenzoylated maltose and lactose (Table 2, entries 5 and 6) did not proceed at room temperature and required heating at 55 °C for 2 h to provide the desired products β-D-maltopyranosyl azide and β-D-lactopyranosyl azide **14** and **15** in 91% and 84% yields, respectively. We found these results very intriguing as the rate of *N*-glycosylation of benzoylated glycosyl donors which is usually considered low, could be achieved using a catalytic amount of the mildly Lewis acidic AuBr_3_ and an excellent azide source, trimethylsilyl azide.

**Table 2:** Scope of AuBr_3_-catalyzed azido glycosylation of perbenzoylated sugars.

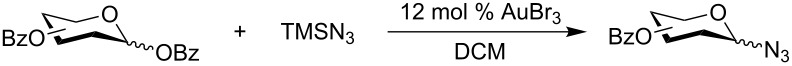				

Entry^a^	Substrate	Product	Time (h)/ temp	α:β ratio/ yield^b^

1	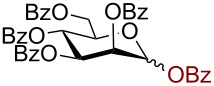	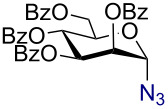 **10**	3/rt	α:β 49:1 α:90%
2	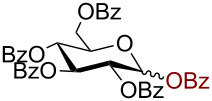	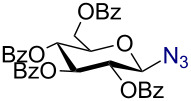 **11**	3/rt	α:β 1:49 β:88%
3	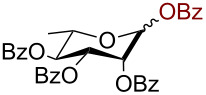	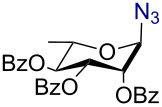 **12**	1/rt	α:β 9:1/ α:71%
4	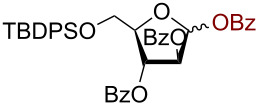	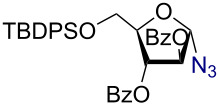 **13**	1/rt	α only 70%
5	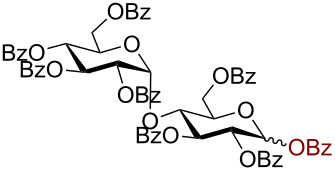	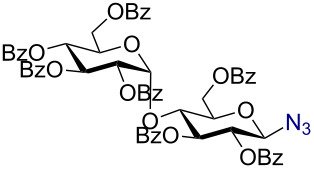 **14**	2/55 °C	β only 91%
6	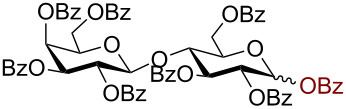	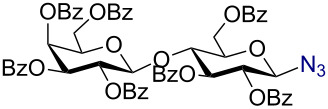 **15**	2.5/55 °C	β only 84%

^a^All reactions were carried out on a 300 mg scale using 12 mol % AuBr_3_ and 3 equiv TMSN_3_ in 4 mL of CH_2_Cl_2_; rt: room temperature; ^b^isolated purified yield.

Further, we checked the possibility of *O*-glycosylation and *C*-glycosylation of peracetylated sugars with 10 mol % AuBr_3_, but the starting materials remained unaffected. Finally, the potential of the gold(III)-catalyzed azidation for large scale applications was demonstrated by performing a gram-scale synthesis on glucose peracetate giving product **2** in 90% yield.

## Conclusion

In summary, a facile methodology demonstrating the ability of Au(III) in catalyzing the azidation of deactivated sugars was shown. The reaction proceeds in the absence of molecular sieves without forming lactols as byproducts. This operationally simple protocol enables the synthesis of various *N*-glycoconjugates offering a wide range of applications and further demonstrates the value of gold catalysis in carbohydrate chemistry.

## Experimental

**General experimental methods:** Chemicals and materials were obtained from commercial sources and used without further purification unless otherwise noted. ^1^H and ^13^C NMR spectra were recorded on a 400 MHz and 100 MHz spectrometer, respectively using CDCl_3_ as the solvent. Chemical shifts (δ) are given in ppm. For perbenzoate compounds **10**–**15**, tetramethyl silane was used as internal standard. Electrospray ionization (ESI) was used for high resolution mass spectrometry (HRMS). An FTIR spectrometer was used for recording IR spectra and only major peaks are reported in cm^−1^. Optical rotations were measured on a polarimeter using sodium light (D line at 589 nm). Column chromatography was performed on silica gel (120–200 mesh) using mixtures of ethyl acetate and hexane as the eluents.

**General procedure for the anomeric azidation:** To a solution of peracetylated or perbenzoylated sugars (300 mg) in 4 mL of dry DCM at room temperature, TMSN_3_ (3 equiv) was added followed by the addition of AuBr_3_ (amounts of the catalyst are given in Table 1 and Table 2). The reaction mixture was stirred either at room temperature or heated to 55–60 °C as mentioned in the Table 1 and Table 2. Then, the reaction was quenched by adding triethylamine (20 μL). The mixture was concentrated in vacuo and the crude product was purified by column chromatography. Alternatively, the reaction can be quenched by adding sodium bicarbonate solution followed by extraction with DCM (2 × 20 mL). The combined organic layers were washed with water, brine and dried over Na_2_SO_4_ and concentrated to dryness. The residue was purified by column chromatography on silica gel using petroleum ether (bp 60–70 °C) and EtOAc.

## Supporting Information

File 1 Plausible catalytic cycle, experimental data and copies of ^1^H and ^13^C NMR spectra of glycosyl azides **1–15** were provided.
