# Supplementary material for: AuBr3-catalyzed azidation of per-O-acetylated and per-O-benzoylated sugars
Source: Beilstein J Org Chem. 2018 Mar 22;14:682–7. doi: 10.3762/bjoc.14.56 (PMC5870170; doi:10.3762/bjoc.14.56)
Supplement: File 1 — Plausible catalytic cycle, experimental data and copies of 1H and 13C NMR spectra of glycosyl azides 1–15 were provided. [file Beilstein_J_Org_Chem-14-682-s001.pdf]

Supporting Information
for
$AuBr_3$-catalyzed azidation of per-*O*-acetylated and per-*O*-benzoylated sugars

Jayashree Rajput, Srinivas Hotha and Madhuri Vangala*

Address: Department of Chemistry, Indian Institute of Science Education and Research, Pune 411 008, India

Email: Madhuri Vangala* - madhuri@iiserpune.ac.in

*Corresponding author

Plausible catalytic cycle, experimental data and copies of ^{1}H and ^{13}C NMR spectra of glycosyl azides 1–15 were provided

Contents..........Page

Plausible catalytic cycle S2

Experimental data of compounds **1–15** S3

References S8

^{1}H, ^{13}C, NMR spectra of compounds **1–9** S9

^{1}H, ^{13}C, NMR spectra of compounds **10–15** S27

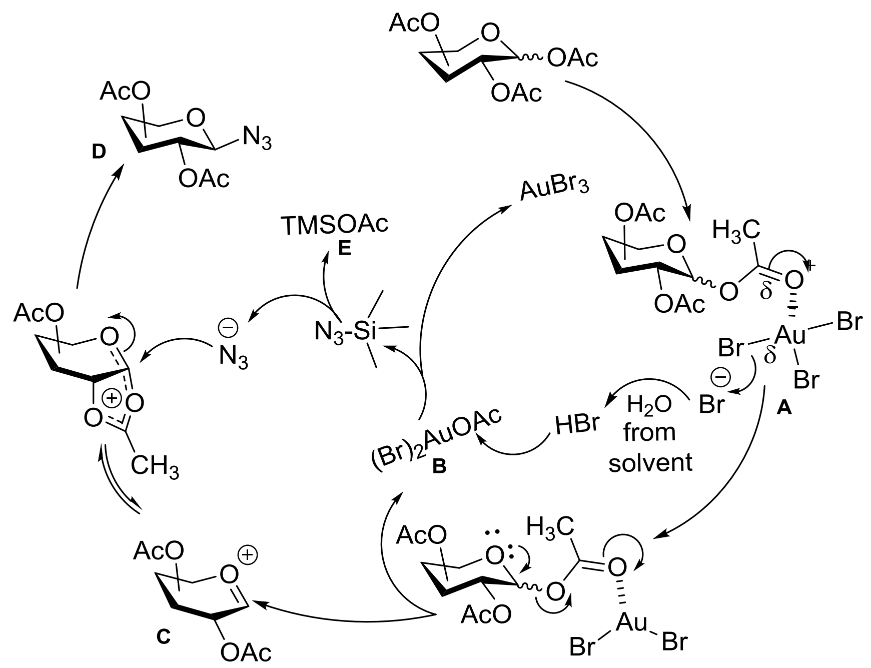

Figure S1: Plausible mechanism for $AuBr_3$-catalyzed azido glycosidation

Experimental data of compounds 1–15

2,3,4,6-Tetra-*O*-acetyl-β-D-glucopyranosyl azide (1).[1] White solid: R_f=0.5(EtOAc:hexane 45:55); m.p. 124-125 °C (lit. m.p. 127 °C); $[\alpha]_D^{25}$ -33.0 (*c* 0.53, $CHCl_3$); IR ($CHCl_3$) *v* 2113, 1747, 1214, 1036 cm^{-1}; ^1H NMR (400 MHz, $CDCl_3$) δ 5.22 (t, *J* = 9.5 Hz, 1H), 5.10 (t, *J* = 9.7 Hz, 1H), 4.95 (t, *J* = 9.2 Hz, 1H), 4.64 (d, *J* = 8.9 Hz, 1H), 4.27 (dd, *J* = 12.5, 4.8 Hz, 1H), 4.17 (dd, *J* = 12.5, 2.3 Hz, 1H), 3.79 (ddd, *J* = 10.0, 4.8, 2.3 Hz, 1H), 2.10 (s, 3H), 2.08 (s, 3H), 2.03 (s, 3H), 2.01 (s, 3H); ^{13}C NMR (100 MHz, $CDCl_3$) δ 170.6, 170.1, 169.3, 169.2, 87.9, 74.1, 72.6, 70.7, 67.9, 61.7, 20.7, 20.5 (3 X $COCH_3$); HRMS (TOF): $[M + Na]^+$ calcd for $C_{14}H_{19}O_9N_3Na$ 396.1019, found 396.1022.

2,3,4,6-Tetra-*O*-acetyl-β-D-galactopyranosyl azide (2).[1] White solid: R_f=0.4(EtOAc:hexane 35:65); m.p. 93-95 °C (lit. m.p. 95-97 °C); $[\alpha]_D^{25}$ -16.2 (*c* 0.34, $CHCl_3$); IR ($CHCl_3$) *v* 2114, 1742, 1208, 1049 cm^{-1}; ^1H NMR (400 MHz, $CDCl_3$) δ 5.41 (dd, *J* = 3.2, 0.7 Hz, 1H), 5.15 (dd, *J* = 10.4, 8.7 Hz, 1H), 5.02 (dd, *J* = 10.3, 3.4 Hz, 1H), 4.59 (d, *J* = 8.7 Hz, 1H), 4.20 – 4.11 (m, 2H), 4.03-3.98 (m, 1H), 2.16 (s, 3H), 2.08 (s, 3H), 2.05 (s, 3H), 1.98 (s, 3H); ^{13}C NMR (100 MHz, $CDCl_3$) δ 170.5, 170.2, 170.1, 169.5, 88.4, 73.0, 70.8, 68.1, 66.9, 61.3, 20.8, 20.7, 20.7, 20.6; HRMS (TOF): $[M + Na]^+$ calcd for $C_{14}H_{19}O_9N_3Na$ 396.1019, found 396.1032.

2,3,4,6-Tetra-*O*-acetyl-α-D-mannopyranosyl azide (3).[1] Thick liquid: R_f=0.5(EtOAc:hexane 35:65); $[\alpha]_D^{25}$ +124.3 (*c* 0.41, $CHCl_3$); IR ($CHCl_3$) *v* 2116, 1742, 1207, 1045 cm^{-1}; ^1H NMR (400 MHz, $CDCl_3$) δ 5.38 (d, *J* = 1.8 Hz, 1H), 5.30 – 5.21 (m, 2H), 5.14 (dd, *J* = 2.8, 2.0 Hz, 1H), 4.29 (dd, *J* = 12.4, 5.5 Hz, 1H), 4.18-4.11 (m, 2H), 2.15 (s, 3H), 2.10 (s, 3H), 2.04 (s, 3H), 1.98 (s, 3H); ^{13}C NMR (100 MHz, $CDCl_3$) δ 170.7, 169.9, 169.8, 169.7, 87.6, 70.7, 69.2, 68.3, 65.7, 62.2, 20.9, 20.8, 20.7, 20.7; HRMS (TOF): $[M + Na]^+$ calcd for $C_{14}H_{19}O_9N_3Na$ 396.1019, found 396.1019.

2,3,5-Tri-*O*-acetyl-β-D-xylopyranosyl azide (4).[1] Thick liquid: R_f=0.4(EtOAc:hexane 25:75); IR ($CHCl_3$) *ν* 2114, 1743, 1204, 1066 cm^{-1}; $[\alpha]_D^{25}$ +92.0 (*c* 0.5, $CHCl_3$); ^1H NMR (400 MHz, $CDCl_3$) δ 5.17 (t, *J* = 8.9 Hz, 1H), 4.96 (td, *J* = 9.2, 5.3 Hz, 1H), 4.85 (t, *J* = 8.5 Hz, 1H), 4.62 (d, *J* = 8.1 Hz, 1H), 4.19 (dd, *J* = 11.7, 5.3 Hz, 1H), 3.42 (dd, *J* = 11.7, 9.6 Hz, 1H), 2.06 (s, 3H), 2.03 (s, 3H), 2.02 (s, 3H); ^{13}C NMR (100 MHz, $CDCl_3$) 170.1, 169.8, 169.4, 88.4, 71.6, 70.4, 68.5, 64.3, 20.7, 20.7, 20.6; HRMS (TOF): $[M + Na]^+$ calcd for $C_{11}H_{15}O_7N_3Na$ 324.0808, found 324.0813.

2,3,4-Tri-*O*-acetyl-β-L-fucopyranosyl azide (5). White solid: R_f=0.5(EtOAc:hexane 30:70); m.p. 101-103 °C; $[\alpha]_D^{25}$ +35.3 (*c* 0.77, $CHCl_3$); IR ($CHCl_3$) *ν* 2111, 1742, 1210, 1055 cm^{-1}; ^1H NMR (400 MHz, $CDCl_3$) δ(major isomer) 5.26 (d, *J* = 3.0 Hz, 1H), 5.13 (dd, *J* = 10.3, 8.7 Hz, 1H), 5.02 (dd, *J* = 10.3, 3.4 Hz, 1H), 4.57 (d, *J* = 8.7 Hz, 1H), 3.93 – 3.86 (m, 1H), 2.18 (s, 3H), 2.07 (s, 3H), 1.98 (s, 3H), 1.25 (d, *J* = 6.4 Hz, 3H); ^{13}C NMR (100 MHz, $CDCl_3$) δ 170.6, 170.2, 169.6, 88.3, 71.6, 71.3, 70.1, 68.3, 20.8, 20.7, 20.7, 16.1; HRMS (TOF): $[M + Na]^+$ calcd for $C_{12}H_{17}O_7N_3Na$ 338.0964, found 338.0968.

2,3,6-Tri-*O*-acetyl-4-*O*-(2,3,4,6-tetra-*O*-acetyl-β-D-glucopyranosyl)-β-D-glucopyranosyl azide (6). White solid: R_f=0.6(EtOAc:hexane 25:75); mp- 170-172 ºC; $[\alpha]_D^{25}$ -26.6 (*c* 0.42, $CHCl_3$); IR ($CHCl_3$) *ν* 2113, 1746, 1215, 1038 cm^{-1}; ^1H NMR (400 MHz, $CDCl_3$) δ 5.20 – 5.08 (m, 2H), 5.04 (t, *J* = 9.6 Hz, 1H), 4.93 – 4.81 (m, 2H), 4.60 (d, *J* = 8.8 Hz, 1H), 4.54-4.47 (m, 2H), 4.36 (dd, *J* = 12.5, 4.4 Hz, 1H), 4.10 (dd, *J* = 12.2, 5.0 Hz, 1H), 4.02 (dd, *J* = 12.5, 2.1 Hz, 1H), 3.82 – 3.74 (m, 1H), 3.72 – 3.61 (m, 2H), 2.12 (s, 3H), 2.07 (s, 3H), 2.05 (s, 3H), 2.01 (s, 3H), 2.00 (s, 3H), 1.99 (s, 3H), 1.96 (s, 3H); ^{13}C NMR (100 MHz, $CDCl_3$) δ 170.5, 170.3, 170.3, 169.7, 169.5, 169.4, 169.1, 100.9, 87.8, 76.1, 74.9, 72.9, 72.3, 72.1, 71.6, 70.9, 67.8, 61.7, 61.6, 20.9, 20.7, 20.6, 20.6, 20.5; HRMS (TOF): $[M + Na]^+$ calcd for $C_{26}H_{35}O_{17}N_3Na$ 684.1864, found 684.1804.

2,3,4,6-Tetra-*O*-acetyl-α-D-glucopyranosyl-(1-4)-2,3,6-tri-*O*-acetyl-α-D-glucopyranosyl-(1-4)-2,3,6-tri-*O*-acetyl-β-D-glucopyranosyl azide (7).[1] White solid: R_f=0.3(EtOAc:hexane 25:75); mp- 96-98 ºC (lit. m.p. 95-96 °C); $[\alpha]_D^{25}$ +65.8 (*c* 0.3, $CHCl_3$); IR ($CHCl_3$) *v* 2116, 1742, 1214, 1028 cm^{-1}; ^1H NMR (400 MHz, $CDCl_3$) δ 5.41 – 5.33 (m, 3H), 5.32-5.23 (m, 2H), 5.06 (t, *J* = 9.9 Hz, 1H), 4.84 (dd, *J* = 10.5, 4.0 Hz, 1H), 4.80 – 4.68 (m, 3H), 4.48 (ddd, *J* = 14.8, 12.4, 2.4 Hz, 2H), 4.31 (dd, *J* = 12.3, 4.3 Hz, 1H), 4.24 (dd, *J* = 12.5, 3.5 Hz, 1H), 4.17 (dd, *J* = 12.3, 3.2 Hz, 1H), 4.04 (dd, *J* = 12.5, 2.2 Hz, 1H), 4.01 – 3.89 (m, 4H), 3.84 – 3.78 (m, 1H), 2.18 (s, 3H), 2.15 (s, 3H), 2.09 (s, 3H), 2.04 (s, 3H), 2.04 (s, 3H), 2.02 (s, 3H), 2.01 (s, 3H), 1.99 (s, 9H, 3 × $COCH_3$); ^{13}C NMR (100 MHz, $CDCl_3$) δ 170.7, 170.7, 170.6, 170.5, 170.1, 170.0, 169.8, 169.6, 169.5, 96.0, 95.8, 87.5,75.0, 74.3, 73.6, 72.5, 71.8, 71.6, 70.5, 70.2, 69.5, 69.2, 68.6, 67.9, 62.8, 62.4, 61.5, 21.0, 20.9, 20.8, 20.7, 20.6; HRMS (TOF): $[M + Na]^+$ calcd for $C_{38}H_{51}N_3NaO_{25}$ 972.2709, found 972.2708.

2,3,5-Tri-*O*-acetyl-β-D-ribofuranosyl azide (8).[1] Thick liquid: R_f=0.6(EtOAc:hexane 25:75); IR ($CHCl_3$) *v* 2110, 1743, 1208, 1051 cm^{-1}; ^1H NMR (400 MHz, $CDCl_3$) δ 5.35 (d, *J* = 2.0 Hz, 1H), 5.32 (dd, *J* = 6.8, 4.9 Hz, 1H), 5.13 (dd, *J* = 4.8, 2.0 Hz, 1H), 4.41 (dd, *J* = 12.2, 3.2 Hz, 1H), 4.36 – 4.32 (m, 1H), 4.14 (dd, *J* = 12.2, 4.3 Hz, 1H), 2.12 (s, 3H), 2.11 (s, 3H), 2.06 (s, 3H); ^{13}C NMR (100 MHz, $CDCl_3$) δ 170.6, 169.6, 169.5, 92.8, 79.5, 74.6, 70.6, 63.1, 20.7, 20.6, 20.5; HRMS (TOF): $[M + H]^+$ calcd for $C_{11}H_{16}O_7N_3$ 302.0988, found 302.3091.

3,4,6-Tri-*O*-acetyl-2-*N*-acetyl-2-deoxy-β-D-glucopyranosyl azide (9).[1] White solid: R_f =0.2(EtOAc:hexane 70:30); mp- 153-155 ºC (lit. m.p. 158-161 °C); $[\alpha]_D^{25}$ -28.8 (*c* 0.34, $CHCl_3$); IR ($CHCl_3$) *v* 2114, 1723, 1245, 1091 cm^{-1}; ^1H NMR (400 MHz, $CDCl_3$) δ 5.83 (br s, 1H), 5.25 (t, *J* = 9.6 Hz, 1H), 5.09 (t, *J* = 9.7 Hz, 1H), 4.76 (d, *J* = 9.3 Hz, 1H), 4.26 (dd, *J* = 12.4, 4.8 Hz, 1H), 4.16 (dd, *J* = 12.4, 2.1 Hz, 1H), 3.92 (dt, *J* = 10.4, 9.1 Hz, 1H), 3.79 (ddd, *J* = 10.0, 4.7, 2.2 Hz, 1H), 2.09 (s, 3H), 2.03 (s, 3H), 2.02 (s, 3H), 1.97 (s, 3H); ^{13}C NMR (100 MHz, $CDCl_3$) δ 171.1, 170.8, 170.6, 169.4, 88.5, 74.1, 72.3, 68.2, 62.0, 54.2, 23.3, 20.8, 20.7, 20.7; HRMS (TOF): $[M+H]^+$ calcd for $C_{14}H_{21}O_8N_4$ 373.1359, found 372.9190.

2,3,4,6-Tetra-*O*-benzoyl-α-D-mannopyranosyl azide (10). White solid: R_f=0.6(EtOAc:hexane 25:75); mp- 143-145 ºC; $[\alpha]_D^{25}$ -77.3 (*c* 0.68, $CHCl_3$); IR ($CHCl_3$) *ν* 2105, 1725, 1251, 1045, 701 cm^{-1}; ^1H NMR (400 MHz, $CDCl_3$) δ 8.10 (dd, *J* = 8.0, 0.8 Hz, 2H), 8.02 (dd, *J* = 8.4, 1.2 Hz, 2H), 7.94 (dd, *J* = 8.0, 0.8 Hz, 2H), 7.82 (dd, *J* = 8.1, 1.1 Hz, 2H), 7.62-7.54 (m, 2H), 7.53 – 7.49 (m, 1H), 7.45 – 7.34 (m, 7H), 7.26 (dd, *J* = 10.7, 5.0 Hz, 2H), 6.13 (t, *J* = 10.1 Hz, 1H), 5.81 (dd, *J* = 10.2, 3.2 Hz, 1H), 5.68 (d, *J* = 1.8 Hz, 1H), 5.61 (dd, *J* = 3.2, 2.0 Hz, 1H), 4.75 (dd, *J* = 12.3, 2.4 Hz, 1H), 4.60 (ddd, *J* = 10.0, 4.2, 2.4 Hz, 1H), 4.50 (dd, *J* = 12.3, 4.4 Hz, 1H); ^{13}C NMR (101 MHz, $CDCl_3$) δ ^{13}C NMR (100 MHz, $CDCl_3$) δ 166.2, 165.5, 165.5, 165.4, 133.8, 133.7, 133.4, 133.3, 130.0, 129.9, 129.9, 129.8, 129.0, 128.9, 128.8, 128.8, 128.6, 128.6, 128.5, 87.7, 71.0, 70.2, 69.3, 66.4, 62.6; HRMS (TOF): $[M + Na]^+$ calcd for $C_{34}H_{27}O_9N_3Na$ 644.1645, found 644.1647.

2,3,4,6-Tetra-*O*-benzoyl-β-D-glucopyranosyl azide (11). White solid: R_f=0.4(EtOAc:hexane 15:85); mp- 68-70 ºC; $[\alpha]_D^{25}$ -0.98 (*c* 0.52, $CHCl_3$); IR ($CHCl_3$) *ν* 2113, 1727, 1259, 1094, 708 cm^{-1}; ^1H NMR (400 MHz, $CDCl_3$) δ 8.06-8.03 (m, 2H), 7.98-7.95 (m, 2H), 7.92-7.88 (m, 2H), 7.84-7.80 (m, 2H), 7.58 – 7.47 (m, 3H), 7.44 – 7.24 (m, 9H), 5.93 (td, *J* = 9.6, 0.8 Hz, 1H), 5.72 (t, *J* = 9.9 Hz, 1H), 5.54 – 5.47 (m, 1H), 4.98 (d, *J* = 8.6 Hz, 1H), 4.68 (dd, *J* = 12.3, 3.0 Hz, 1H), 4.52 (dd, *J* = 12.3, 5.1 Hz, 1H), 4.25 (ddd, *J* = 9.6, 5.0, 3.1 Hz, 1H); ^{13}C NMR (100 MHz, $CDCl_3$) δ166.2, 165.8, 165.2, 165.1, 133.8, 133.7, 133.5, 133.4, 130.1, 129.9, 129.9, 129.8, 129.5, 128.8, 128.7, 128.6, 128.5, 128.4, 88.4, 74.6, 72.8, 71.3, 69.2, 62.9; HRMS (TOF): $[M + Na]^+$ calcd for $C_{34}H_{27}O_9N_3Na$ 644.1645, found 644.1647.

2,3,4-Tri-*O*-benzoyl-α-L-rhamnopyranosyl azide (12). White solid: R_f=0.4(EtOAc:hexane 15:85); mp- 142-144 ºC; $[\alpha]_D^{25}$ +68.0 (*c* 0.42, $CHCl_3$); IR ($CHCl_3$) *ν* 2113, 1724, 1243, 1094, 705 cm^{-1}; ^1H NMR (400 MHz, $CDCl_3$) δ 8.05-8.02 (m, 2H), 7.92 – 7.87 (m, 2H), 7.77 – 7.72 (m, 2H), 7.57-7.51 (m, 1H), 7.47 – 7.39 (m, 3H), 7.37-7.28 (m, 3H), 7.17 (t, *J* = 8.0Hz, 2H), 5.69-5.58 (m, 2H), 5.53 (d, *J* = 1.8 Hz, 1H), 5.49 (dd, *J* = 2.9, 2.0 Hz, 1H), 4.33-4.24 (m, 1H), 1.34 (d, *J* = 6.3 Hz, 3H); ^{13}C NMR (100 MHz, $CDCl_3$) δ 165.8, 165.6,

165.5, 133.8, 133.6, 133.4, 130.1, 129.8, 129.8, 129.2, 129.1, 129.0, 128.8, 128.6, 128.43, 87.7, 71.3, 70.6, 69.3, 69.0, 17.8; HRMS (TOF): $[M + Na]^+$ calcd for $C_{27}H_{23}O_7N_3Na$ 524.1434, found 524.1429.

2,3-Di-*O*-benzoyl-5-*O*-tert-butyldiphenylsilyl α-D-arabinofuranosyl azide (13). Thick liquid: R_f=0.6(EtOAc:hexane 10:90); $[\alpha]_D^{25}$ +86.0 (*c* 0.14, $CHCl_3$); IR ($CHCl_3$) *v* 2110, 1709, 1360, 1224, 703 cm^{-1}; ^1H NMR (400 MHz, $CDCl_3$) δ 8.09 (dd, *J* = 7.3, 1.0 Hz, 2H), 7.97 (dd, *J* = 7.3, 0.9 Hz, 2H), 7.73 (dd, *J* = 7.8, 1.3 Hz, 4H), 7.65 – 7.55 (m, 2H), 7.49 (t, *J* = 7.8 Hz, 2H), 7.43 – 7.32 (m, 8H), 5.71 (d, *J* = 4.5 Hz, 1H), 5.64 (s, 1H), 5.38 – 5.36 (m, 1H), 4.55 (q, *J* = 4.6 Hz, 1H), 4.04 (d, *J* = 4.6 Hz, 2H), 1.07 (s, 9H); ^{13}C NMR (100 MHz, $CDCl_3$) δ 165.6, 165.5, 135.8, 135.7, 133.8, 133.7, 133.2, 133.1, 130.1, 129.9, 129.2, 128.8, 128.6, 128.6, 127.9, 94.6, 85.1, 82.2, 77.2, 63.4, 26.9, 19.4; MALDI (TOF): $[M + Na]^+$ calcd for $C_{35}H_{35}N_3NaO_6Si$ 644.2193, found 644.5349.

2,3,6-Tri-*O*-benzoyl-4-*O*-(2,3,4,6-tetra-*O*-benzoyl-α-D-glucoopyranosyl)-β-D-glucopyranosyl azide (14). White solid: R_f=0.7(EtOAc:hexane 30:70); mp- 107-109 ºC; $[\alpha]_D^{25}$ +53.0 (*c* 0.86, $CHCl_3$); IR ($CHCl_3$) *v* 2112, 1725, 1254, 1090, 733 cm^{-1}; ^1H NMR (400 MHz, $CDCl_3$) δ 8.06 – 8.01 (m, 2H), 7.94 – 7.89 (m, 2H), 7.82 – 7.74 (m, 4H), 7.70 – 7.62 (m, 4H), 7.60 – 7.44 (m, 4H), 7.44 – 7.31 (m, 8H), 7.30 – 7.10 (m, 11H), 6.01 (t, *J* = 10.0 Hz, 1H), 5.72 (d, *J* = 9.2 Hz, 1H), 5.68 (d, *J* = 4.2 Hz, 1H), 5.58 (t, *J* = 9.7 Hz, 1H), 5.24 – 5.14 (m, 2H), 4.91 – 4.82 (m, 2H), 4.70 (dd, *J* = 12.3, 4.1 Hz, 1H), 4.48 – 4.30 (m, 3H), 4.20 (dd, *J* = 12.3, 3.7 Hz, 1H), 4.11 (ddd, *J* = 9.5, 3.7, 2.4 Hz, 1H); ^{13}C NMR (100 MHz, $CDCl_3$) δ 166.2, 165.9, 165.7, 165.5, 165.2, 165.2, 165.1, 133.7, 133.6, 133.5, 133.4, 133.2, 130.1, 130.0, 130.0, 129.9, 129.8, 129.7, 129.5, 129.3, 128.9, 128.7, 128.6, 128.5, 128.4, 128.3, 128.3, 128.2, 96.6, 88.0, 75.1, 74.7, 72.7, 71.7, 71.0, 69.9, 69.4, 69.2, 63.2, 62.6; HRMS (TOF): $[M + Na]^+$ calcd for $C_{61}H_{49}O_{17}N_3Na$ 1118.2960, found 1118.2979.

2,3,6-Tri-*O*-benzoyl-4-*O*-(2,3,4,6-tetra-*O*-benzoyl-β-D-galactopyranosyl)-β-D-glucopyranosyl azide (15). White solid: R_f=0.5(EtOAc:hexane 20:80); mp- 132-134 ºC; $[\alpha]_D^{25}$ +35.7 (*c* 0.8, $CHCl_3$); IR ($CHCl_3$) *ν* 2112, 1726, 1261, 1092, 706 cm^{-1}; ^1H NMR (400 MHz, $CDCl_3$) δ 8.08 – 7.96 (m, 10H), 7.92 (dd, *J* = 8.2, 1.0 Hz, 2H), 7.75 (dd, *J* = 8.3, 1.1 Hz, 2H), 7.67 – 7.55 (m, 3H), 7.54 – 7.29 (m, 14H), 7.19 (dt, *J* = 15.7, 7.8 Hz, 4H), 5.86 (t, *J* = 9.5 Hz, 1H), 5.80 – 5.74 (m, 2H), 5.49 – 5.40 (m, 2H), 4.93 (d, *J* = 7.9 Hz, 1H), 4.86 (d, *J* = 8.8 Hz, 1H), 4.65 (dd, *J* = 12.2, 1.6 Hz, 1H), 4.57 (dd, *J* = 12.4, 4.1 Hz, 1H), 4.32 (t, *J* = 9.5 Hz, 1H), 4.00 – 3.89 (m, 2H), 3.77 (d, *J* = 6.7 Hz, 2H); ^{13}C NMR (100 MHz, $CDCl_3$) δ 165.8, 165.6, 165.4, 165.3, 165.3, 165.2, 164.8, 133.6, 133.6, 133.5, 133.4, 133.3, 130.0, 129.9, 129.8, 129.7, 129.6, 129.4, 129.3, 128.8, 128.7, 128.7, 128.6, 128.6, 128.5, 128.4, 128.3, 101.0, 88.2, 75.5, 75.2, 72.6, 71.8, 71.5, 71.1, 69.9, 67.5, 62.1, 61.1; MALDI (TOF): $[M + Na]^+$ calcd for $C_{61}H_{49}O_{17}N_3Na$ 1118.2960, found 1117.2369.

References

[1]Salunke, S. B.; Babu, N. S.; Chen, C.-T. *Chem. Commun.* **2011,** *47*, 10440–10442. doi:10.1039/C1CC13370E

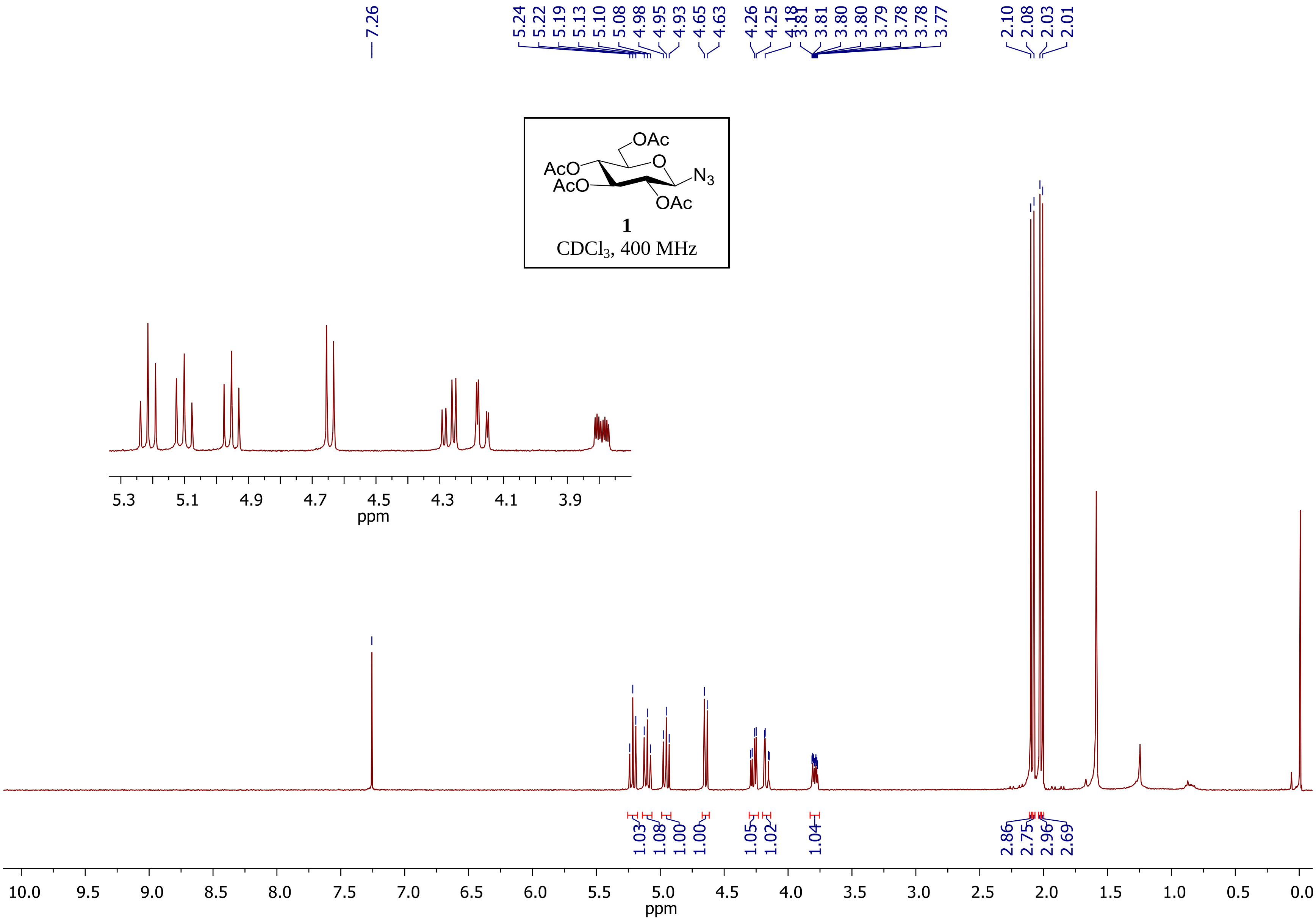
7.26
5.24 5.22 5.19 5.13 5.10 5.08 4.98 4.95 4.93 4.65 4.63 4.26 4.25 4.18 3.81 3.81 3.80 3.80 3.79 3.78 3.78 3.77
2.10 2.08 2.03 2.01
OAc
AcO
AcO
O
N3
OAc
1
CDCl3, 400 MHz
5.3 5.1 4.9 4.7 4.5 4.3 4.1 3.9
ppm
1.03 1.08 1.00 1.00 1.05 1.02 1.04
2.86 2.75 2.96 2.69
10.0 9.5 9.0 8.5 8.0 7.5 7.0 6.5 6.0 5.5 5.0 4.5 4.0 3.5 3.0 2.5 2.0 1.5 1.0 0.5 0.0
ppm

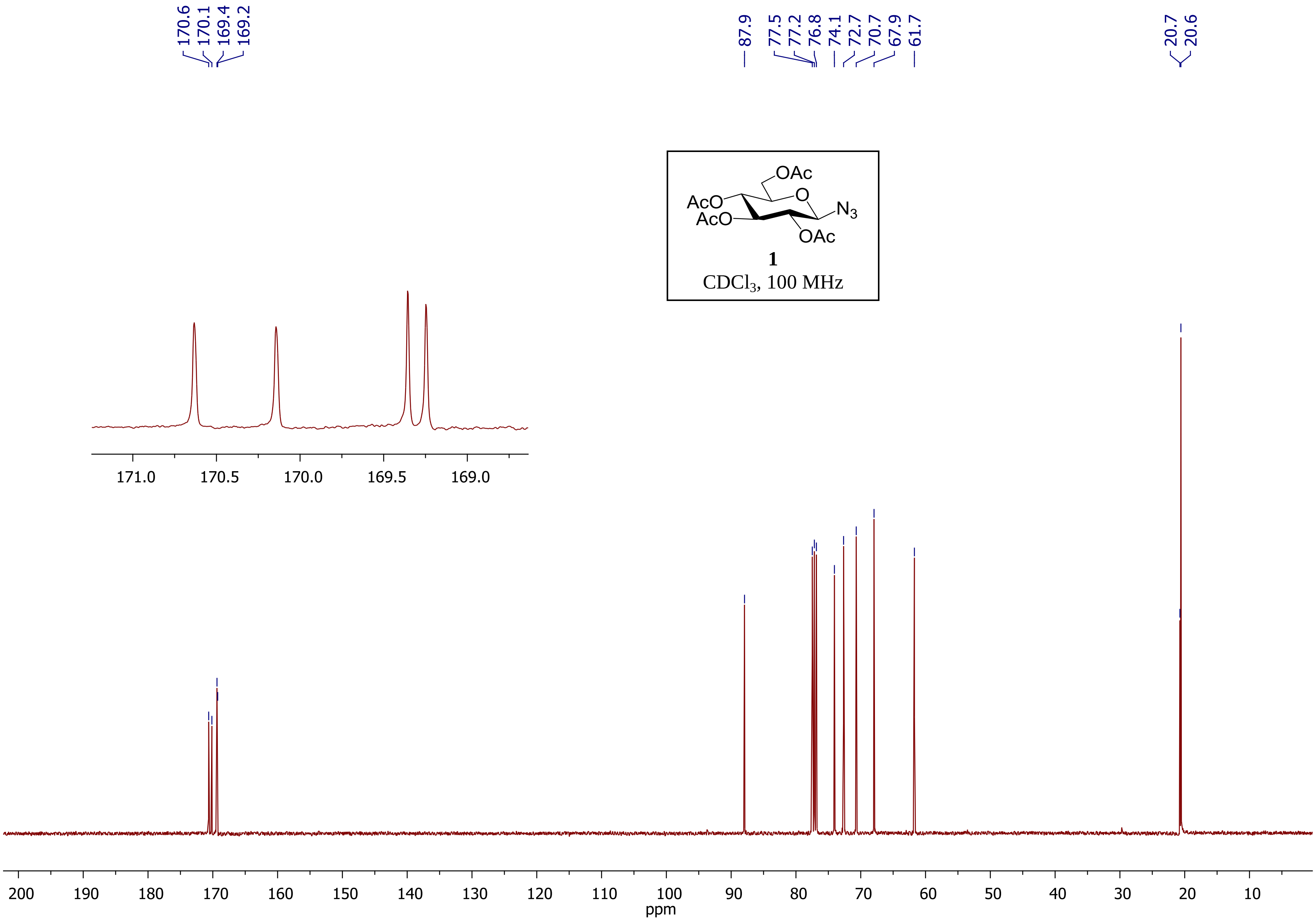
170.6
170.1
169.4
169.2
87.9
77.5
77.2
76.8
74.1
72.7
70.7
67.9
61.7
20.7
20.6
OAc
AcO
AcO
O
N3
OAc
1
CDCl3, 100 MHz
171.0
170.5
170.0
169.5
169.0
200
190
180
170
160
150
140
130
120
110
100
90
80
70
60
50
40
30
20
10
ppm

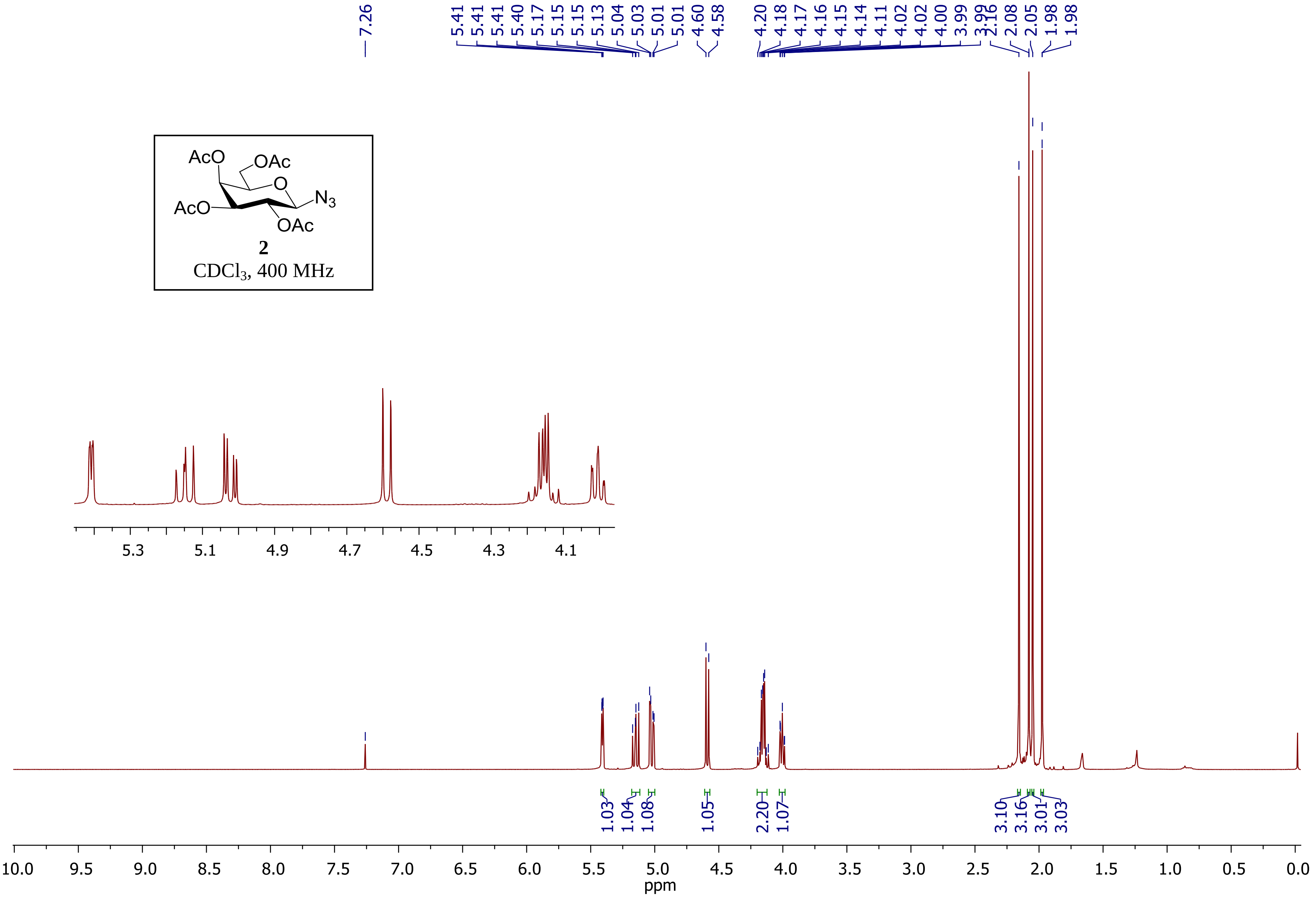

7.26
5.41
5.41
5.41
5.40
5.17
5.15
5.15
5.13
5.04
5.03
5.01
5.01
4.60
4.58
4.20
4.18
4.17
4.16
4.15
4.14
4.11
4.02
4.02
4.00
3.99
3.99
2.16
2.08
2.05
1.98
1.98
AcO
OAc
O
N3
AcO
OAc
2
CDCl3, 400 MHz
5.3
5.1
4.9
4.7
4.5
4.3
4.1
1.03
1.04
1.08
1.05
2.20
1.07
3.10
3.16
3.01
3.03
10.0
9.5
9.0
8.5
8.0
7.5
7.0
6.5
6.0
5.5
5.0
4.5
4.0
3.5
3.0
2.5
2.0
1.5
1.0
0.5
0.0
ppm

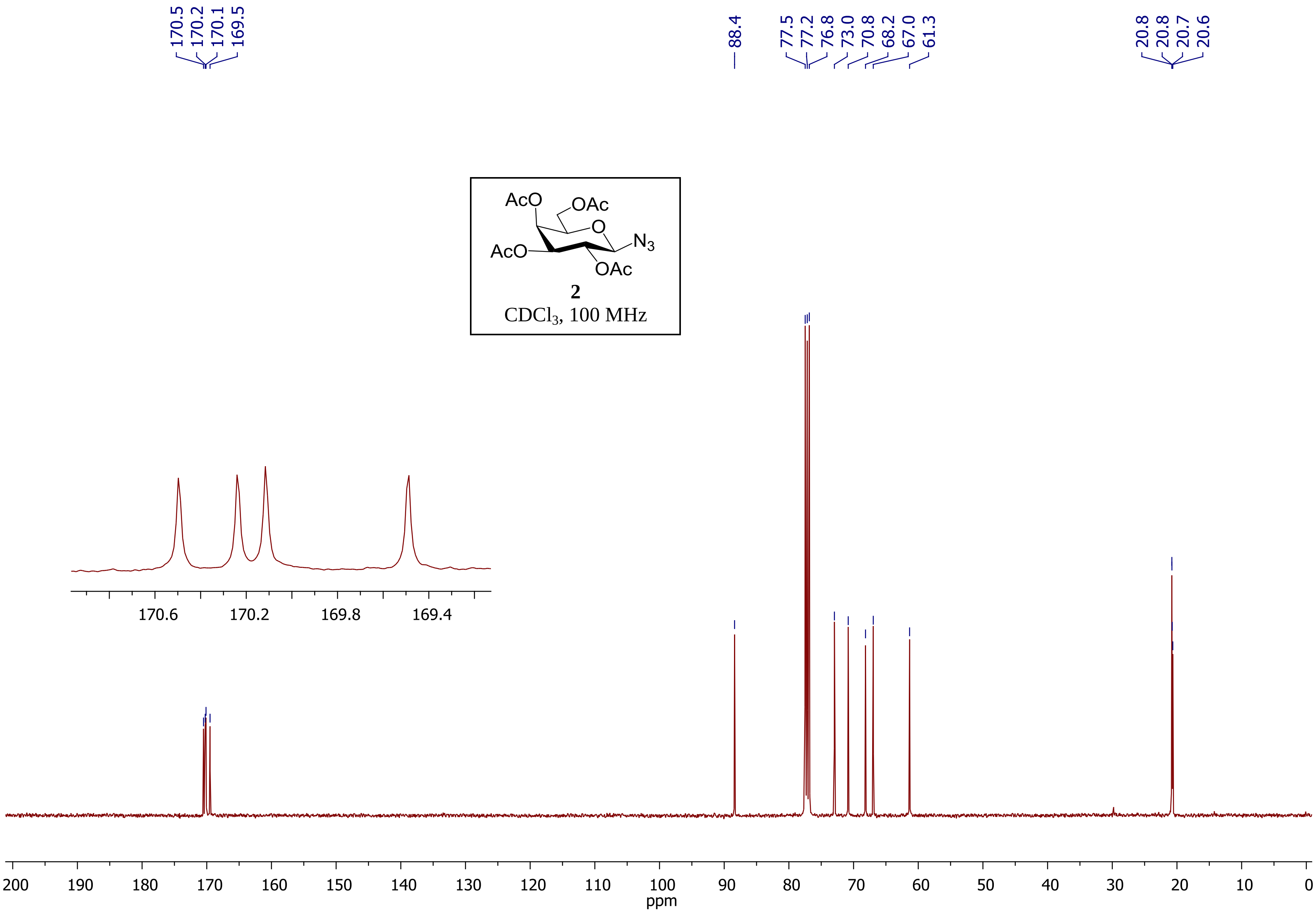

170.5
170.2
170.1
169.5
88.4
77.5
77.2
76.8
73.0
70.8
68.2
67.0
61.3
20.8
20.8
20.7
20.6
AcO
OAc
O
N3
AcO
OAc
2
CDCl3, 100 MHz
170.6
170.2
169.8
169.4
200
190
180
170
160
150
140
130
120
110
100
90
80
70
60
50
40
30
20
10
0
ppm

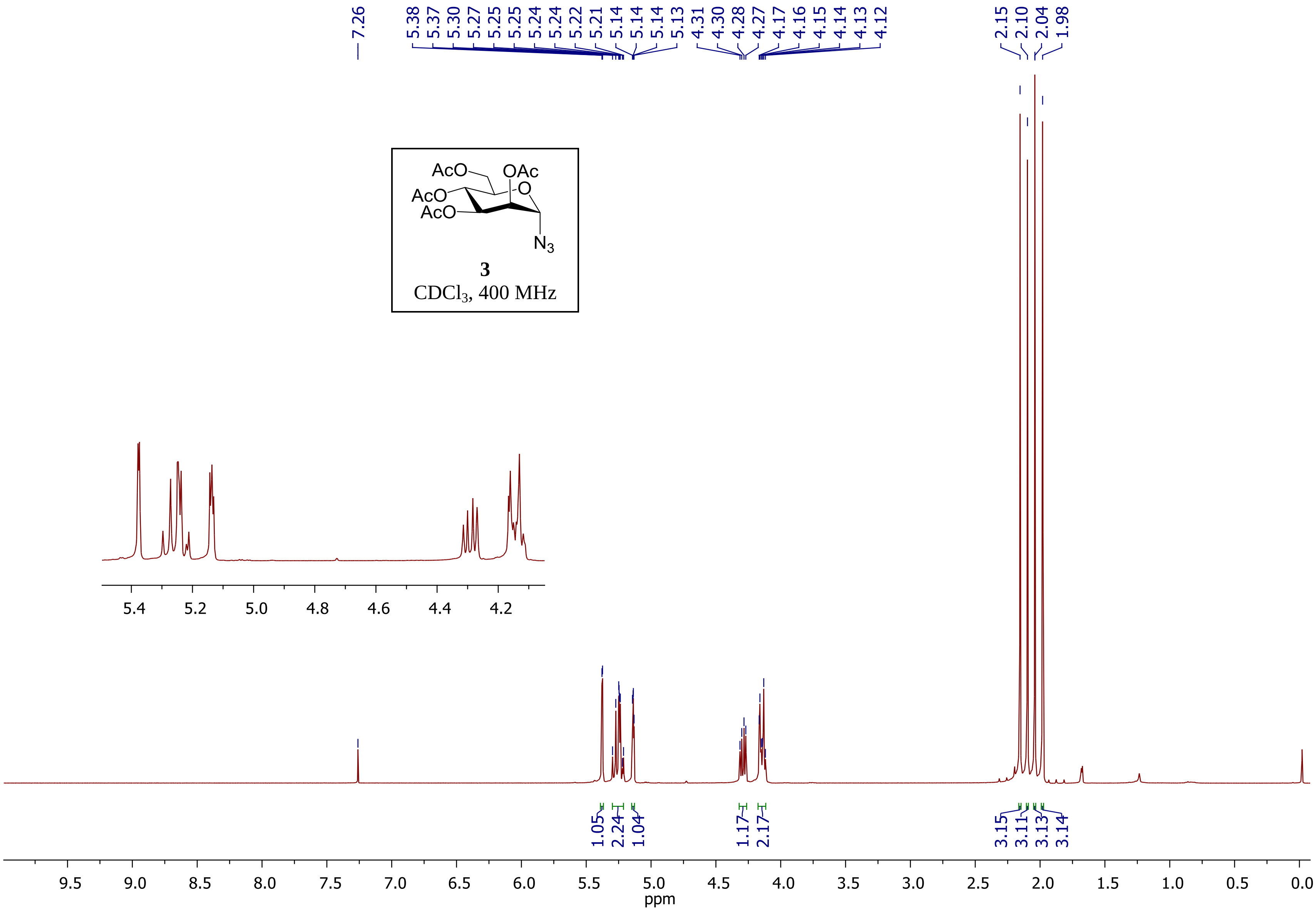

7.26
5.38 5.37 5.30 5.27 5.25 5.25 5.24 5.24 5.22 5.21 5.14 5.14 5.14 5.13 4.31 4.30 4.28 4.27 4.17 4.16 4.15 4.14 4.13 4.12
2.15 2.10 2.04 1.98
AcO
OAc
AcO
AcO
O
N3
3
CDCl3, 400 MHz
5.4 5.2 5.0 4.8 4.6 4.4 4.2
1.05 2.24 1.04 1.17 2.17 3.15 3.11 3.13 3.14
9.5 9.0 8.5 8.0 7.5 7.0 6.5 6.0 5.5 5.0 4.5 4.0 3.5 3.0 2.5 2.0 1.5 1.0 0.5 0.0
ppm

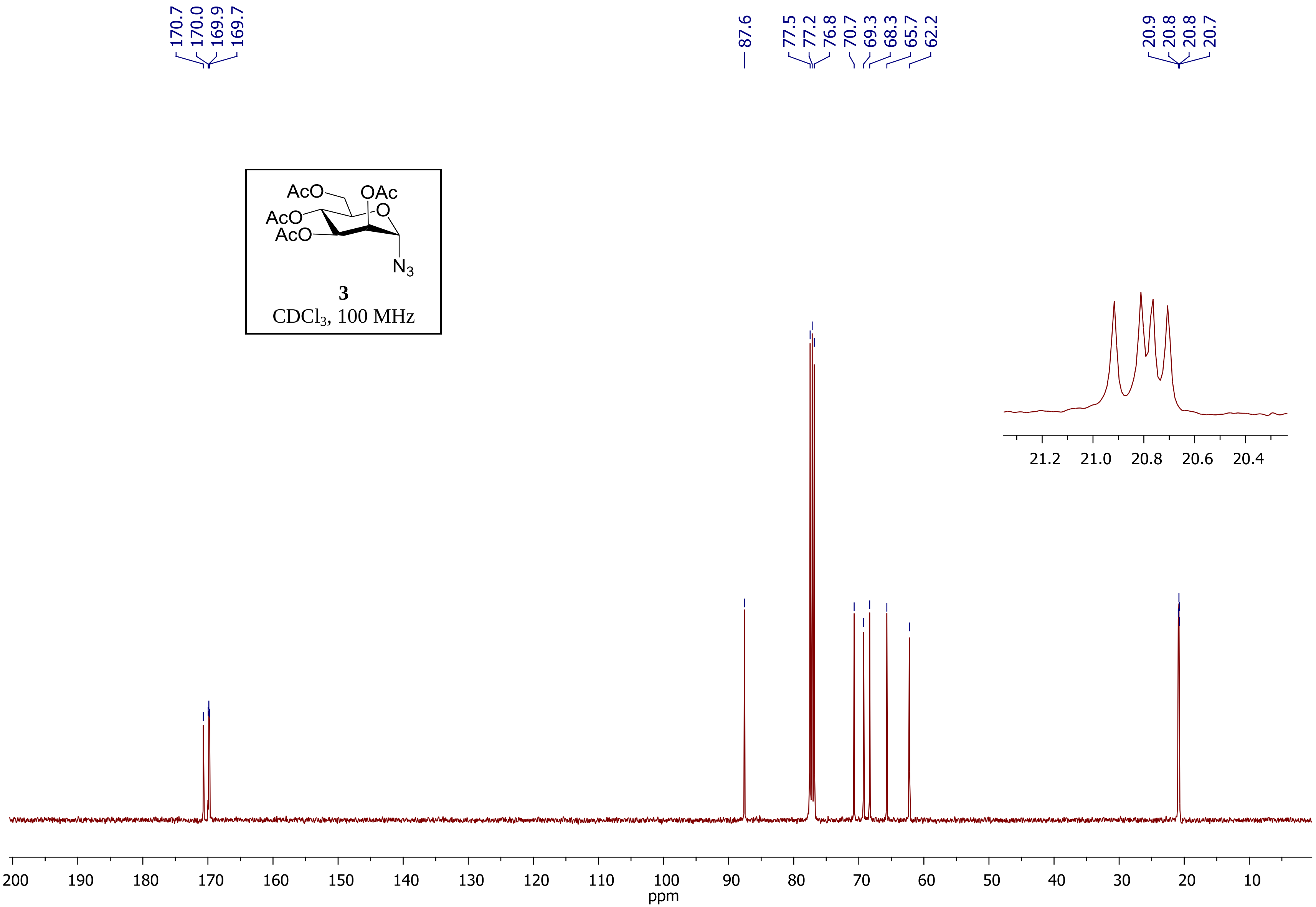

AcO
OAc
O
AcO
AcO
N3
3
CDCl3, 100 MHz
170.7
170.0
169.9
169.7
87.6
77.5
77.2
76.8
70.7
69.3
68.3
65.7
62.2
20.9
20.8
20.8
20.7
21.2
21.0
20.8
20.6
20.4
200
190
180
170
160
150
140
130
120
110
100
90
80
70
60
50
40
30
20
10
ppm

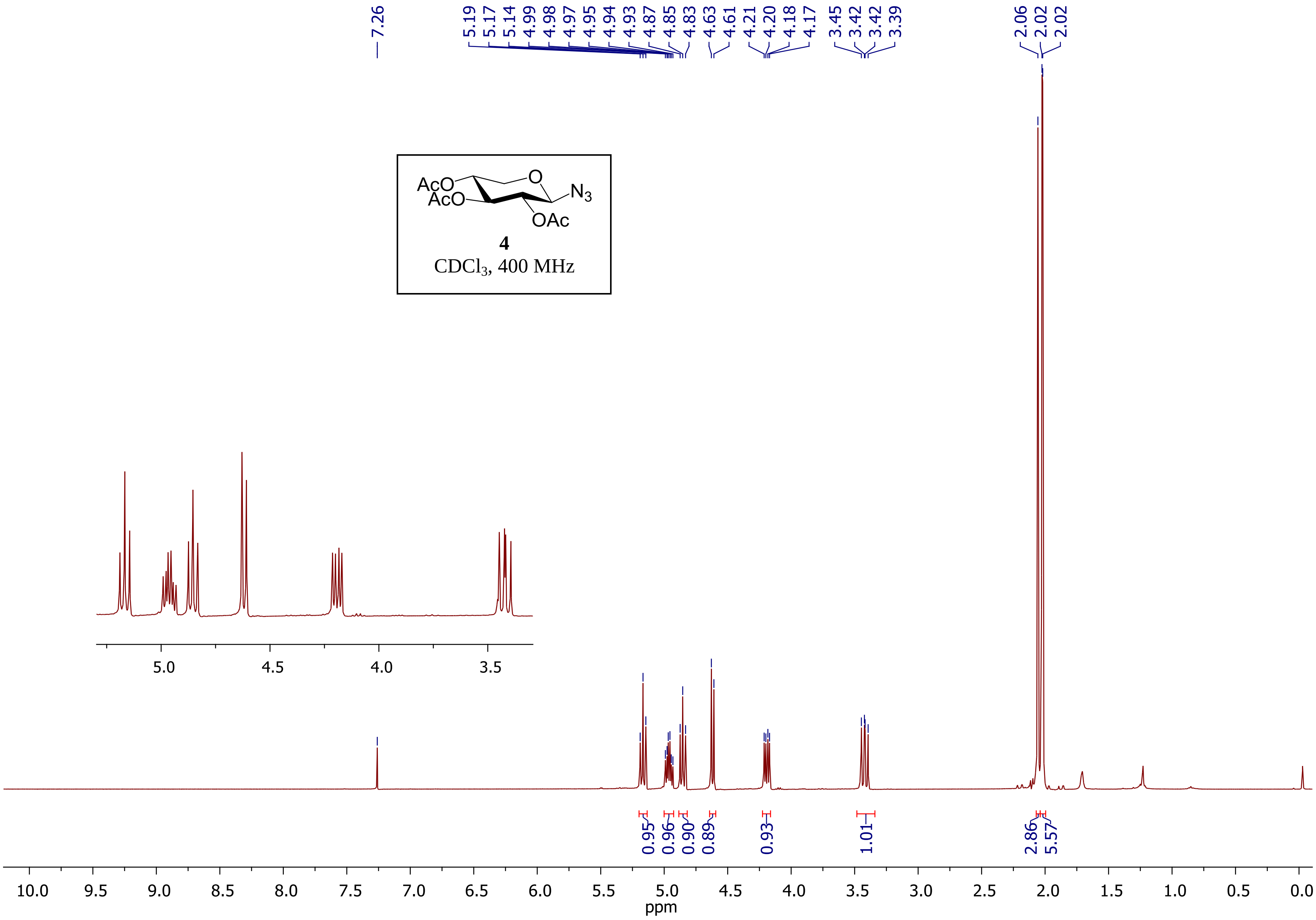
7.26
5.19
5.17
5.14
4.99
4.98
4.97
4.95
4.94
4.93
4.87
4.85
4.83
4.63
4.61
4.21
4.20
4.18
4.17
3.45
3.42
3.42
3.39
2.06
2.02
2.02
AcO
AcO
O
N3
OAc
4
CDCl3, 400 MHz
0.95
0.96
0.90
0.89
0.93
1.01
2.86
5.57
ppm

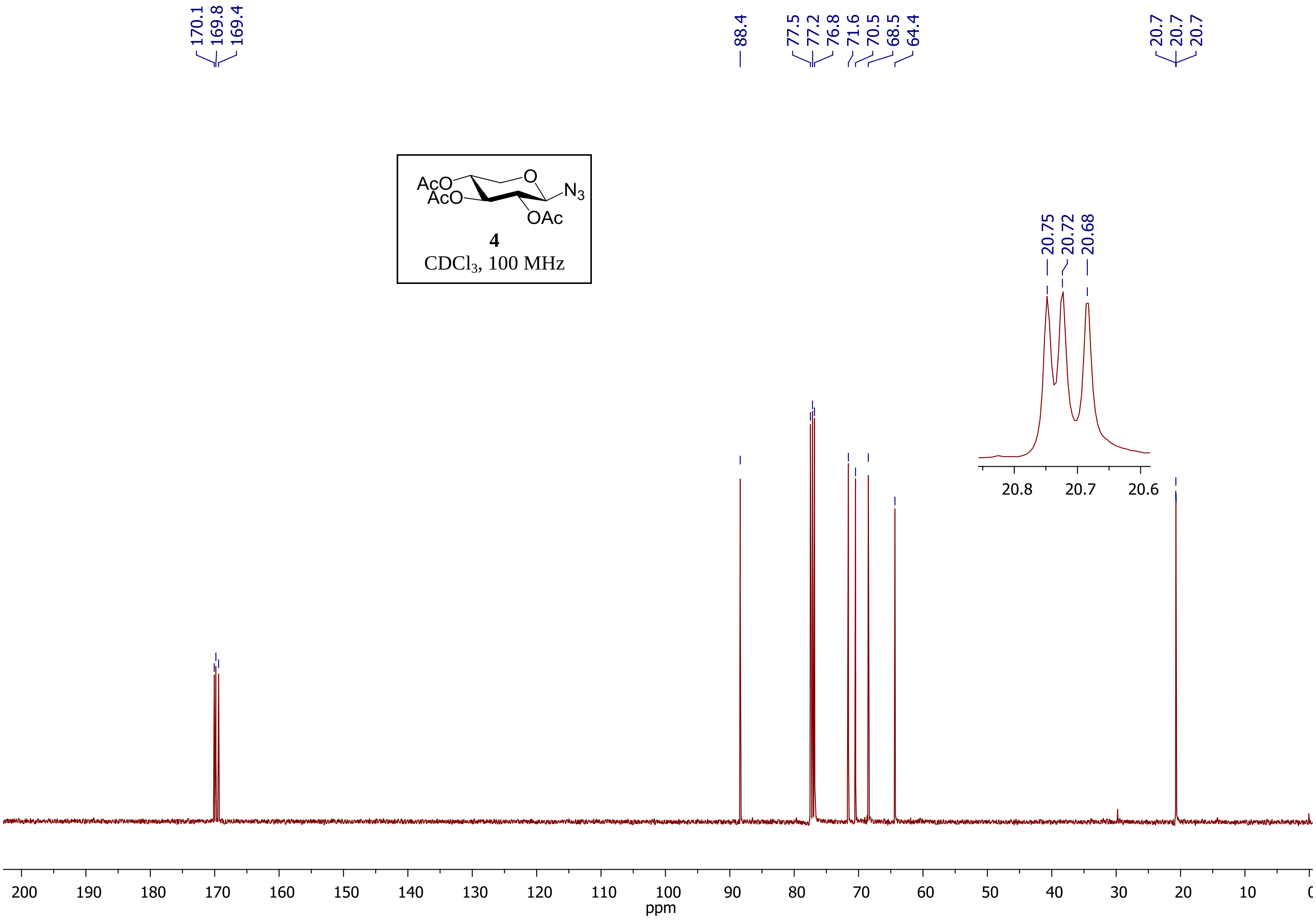
170.1
169.8
169.4
88.4
77.5
77.2
76.8
71.6
70.5
68.5
64.4
20.7
20.7
20.7
AcO
AcO
O
N3
OAc
4
CDCl3, 100 MHz
20.75
20.72
20.68
20.8
20.7
20.6
200
190
180
170
160
150
140
130
120
110
100
90
80
70
60
50
40
30
20
10
ppm

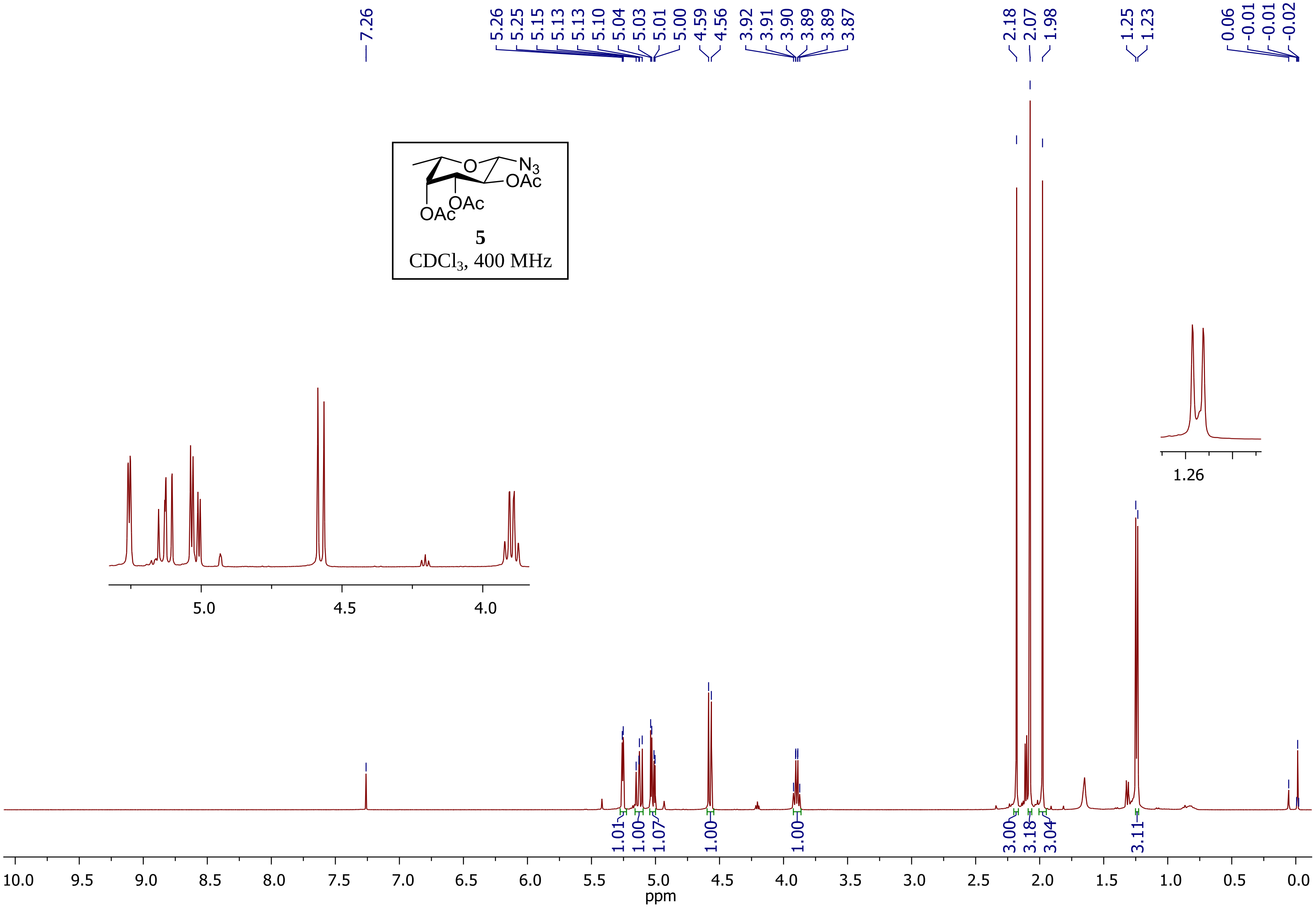

7.26
5.26 5.25 5.15 5.13 5.13 5.10 5.04 5.03 5.01 5.00 4.59 4.56 3.92 3.91 3.90 3.89 3.89 3.87
2.18 2.07 1.98
1.25 1.23
0.06 -0.01 -0.01 -0.02
O
N3
OAc
OAc
OAc
5
CDCl3, 400 MHz
1.26
5.0
4.5
4.0
1.01 1.00 1.07 1.00 1.00 3.00 3.18 3.04 3.11
10.0 9.5 9.0 8.5 8.0 7.5 7.0 6.5 6.0 5.5 5.0 4.5 4.0 3.5 3.0 2.5 2.0 1.5 1.0 0.5 0.0
ppm

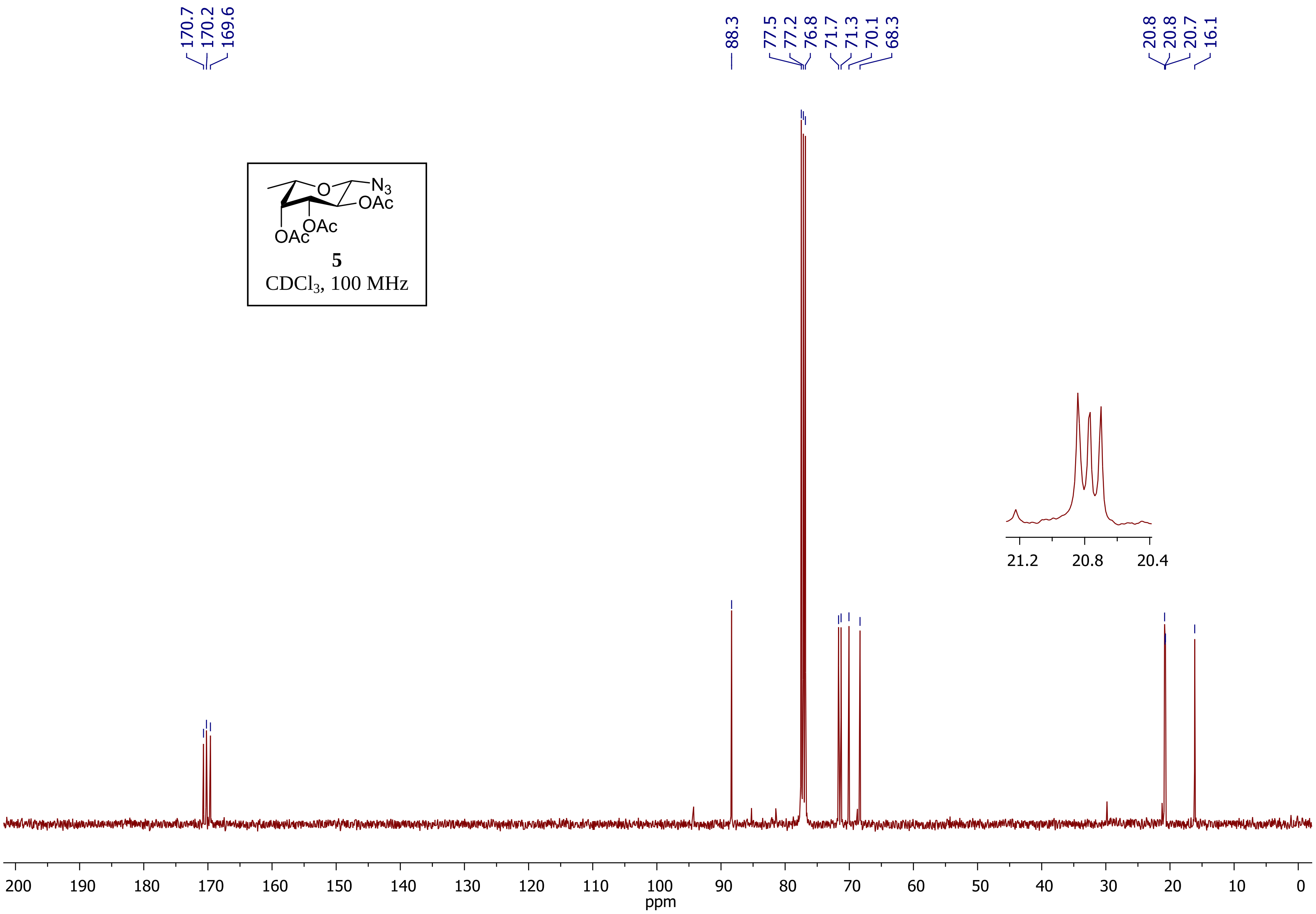
170.7
170.2
169.6
88.3
77.5
77.2
76.8
71.7
71.3
70.1
68.3
20.8
20.8
20.7
16.1
N3
O
OAc
OAc
OAc
5
CDCl3, 100 MHz
21.2
20.8
20.4
200
190
180
170
160
150
140
130
120
110
100
90
80
70
60
50
40
30
20
10
0
ppm

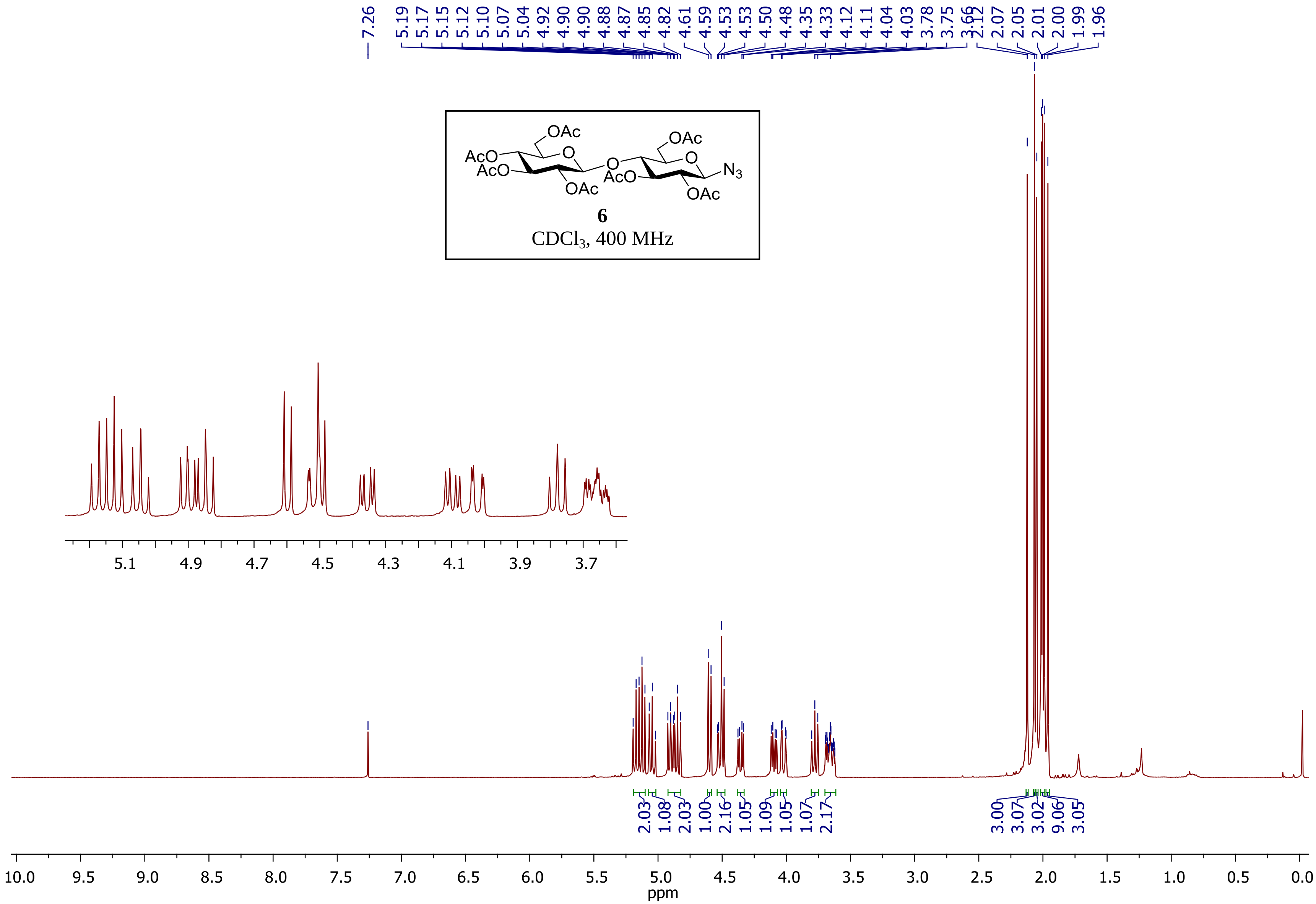
7.26
5.19 5.17 5.15 5.12 5.10 5.07 5.04 4.92 4.90 4.90 4.88 4.87 4.85 4.82 4.61 4.59 4.53 4.53 4.50 4.48 4.35 4.33 4.12 4.11 4.04 4.03 3.78 3.75 3.66 2.12 2.07 2.05 2.01 2.00 1.99 1.96
OAc
AcO
AcO
O
OAc
O
AcO
OAc
O
N3
OAc
6
CDCl3, 400 MHz
2.03 1.08 2.03 1.00 2.16 1.05 1.09 1.05 1.07 2.17
3.00 3.07 3.02 9.06 3.05
ppm

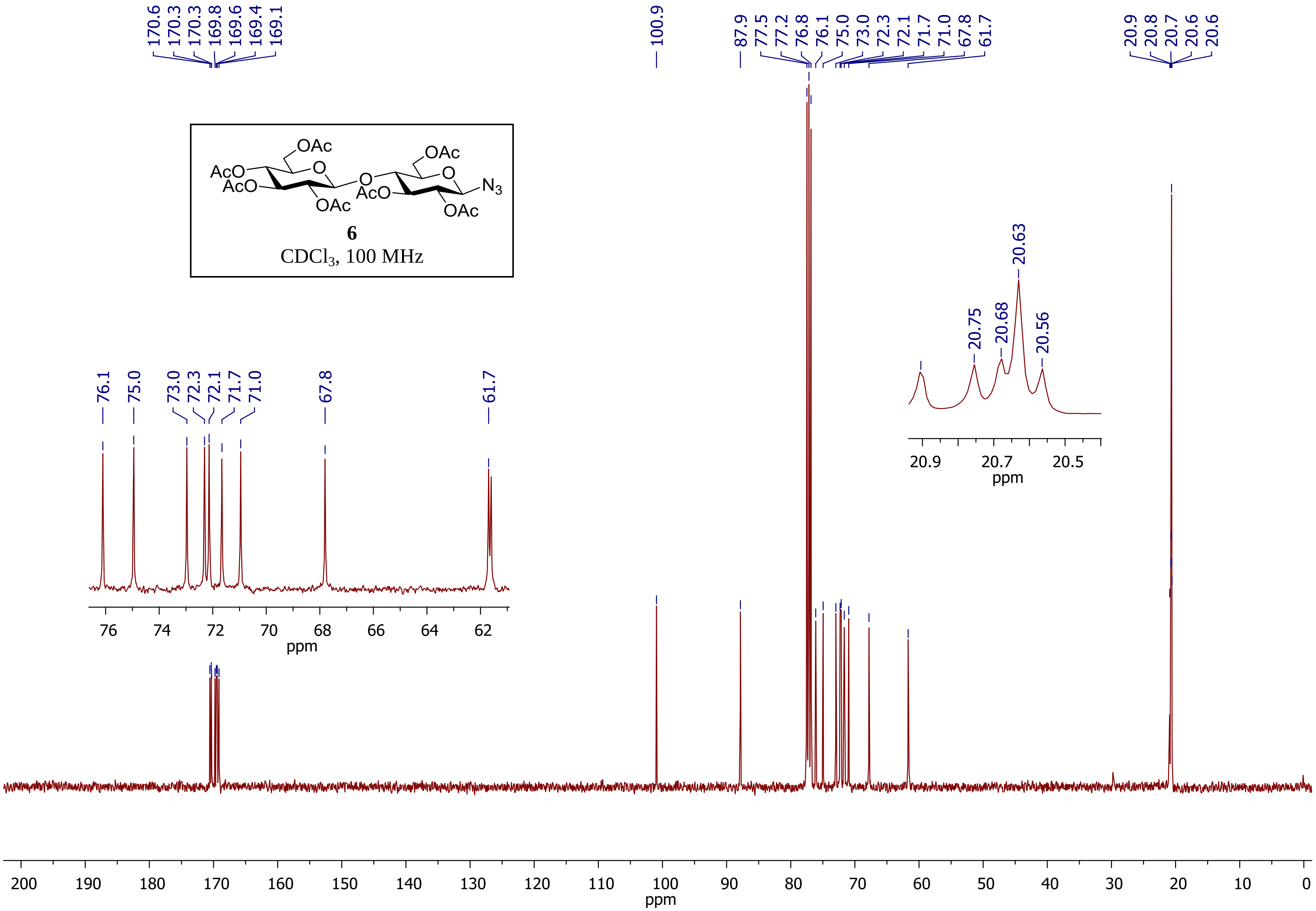

170.6
170.3
170.3
169.8
169.6
169.4
169.1
100.9
87.9
77.5
77.2
76.8
76.1
75.0
73.0
72.3
72.1
71.7
71.0
67.8
61.7
20.9
20.8
20.7
20.6
20.6
OAc
AcO
AcO
O
OAc
O
AcO
OAc
O
N3
OAc
6
CDCl3, 100 MHz
20.75
20.68
20.63
20.56
20.9
20.7
20.5
ppm
76
74
72
70
68
66
64
62
ppm
200
190
180
170
160
150
140
130
120
110
100
90
80
70
60
50
40
30
20
10
0
ppm

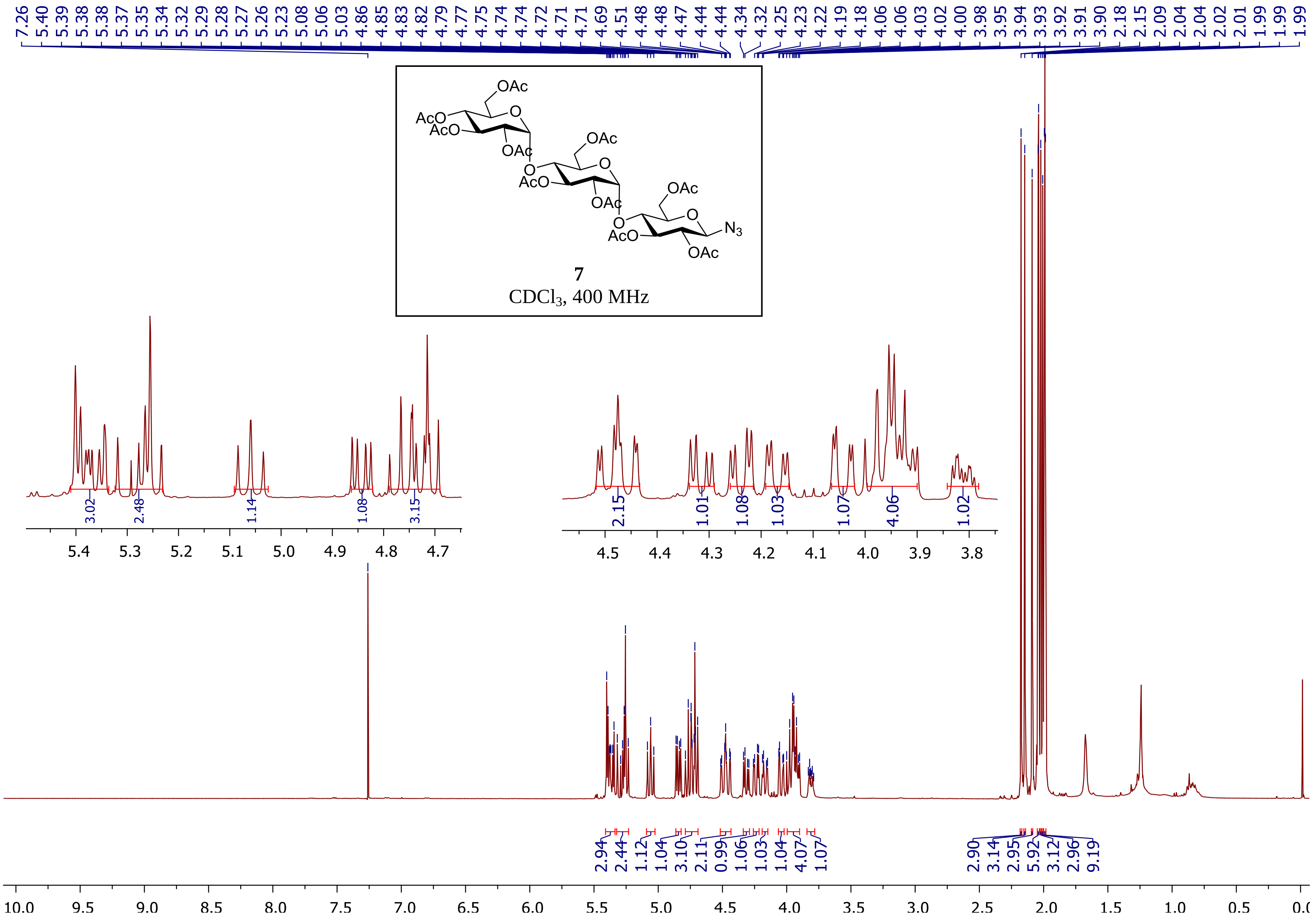
7.26 5.40 5.39 5.38 5.38 5.37 5.35 5.34 5.32 5.29 5.28 5.27 5.26 5.23 5.08 5.06 5.03 4.86 4.85 4.83 4.82 4.79 4.77 4.75 4.74 4.74 4.72 4.71 4.71 4.69 4.51 4.48 4.48 4.47 4.44 4.44 4.34 4.32 4.25 4.23 4.22 4.19 4.18 4.06 4.06 4.03 4.02 4.00 3.98 3.95 3.94 3.93 3.92 3.91 3.90 2.18 2.15 2.09 2.04 2.04 2.02 2.01 1.99 1.99 1.99
OAc
AcO
AcO
O
OAc
O
OAc
AcO
OAc
O
OAc
O
AcO
OAc
N3
7
CDCl3, 400 MHz
3.02
2.48
1.14
1.08
3.15
2.15
1.01
1.08
1.03
1.07
4.06
1.02
2.94
2.44
1.12
1.04
3.10
2.11
0.99
1.06
1.03
1.04
4.07
1.07
2.90
3.14
2.95
5.92
3.12
2.96
9.19
10.0 9.5 9.0 8.5 8.0 7.5 7.0 6.5 6.0 5.5 5.0 4.5 4.0 3.5 3.0 2.5 2.0 1.5 1.0 0.5 0.0

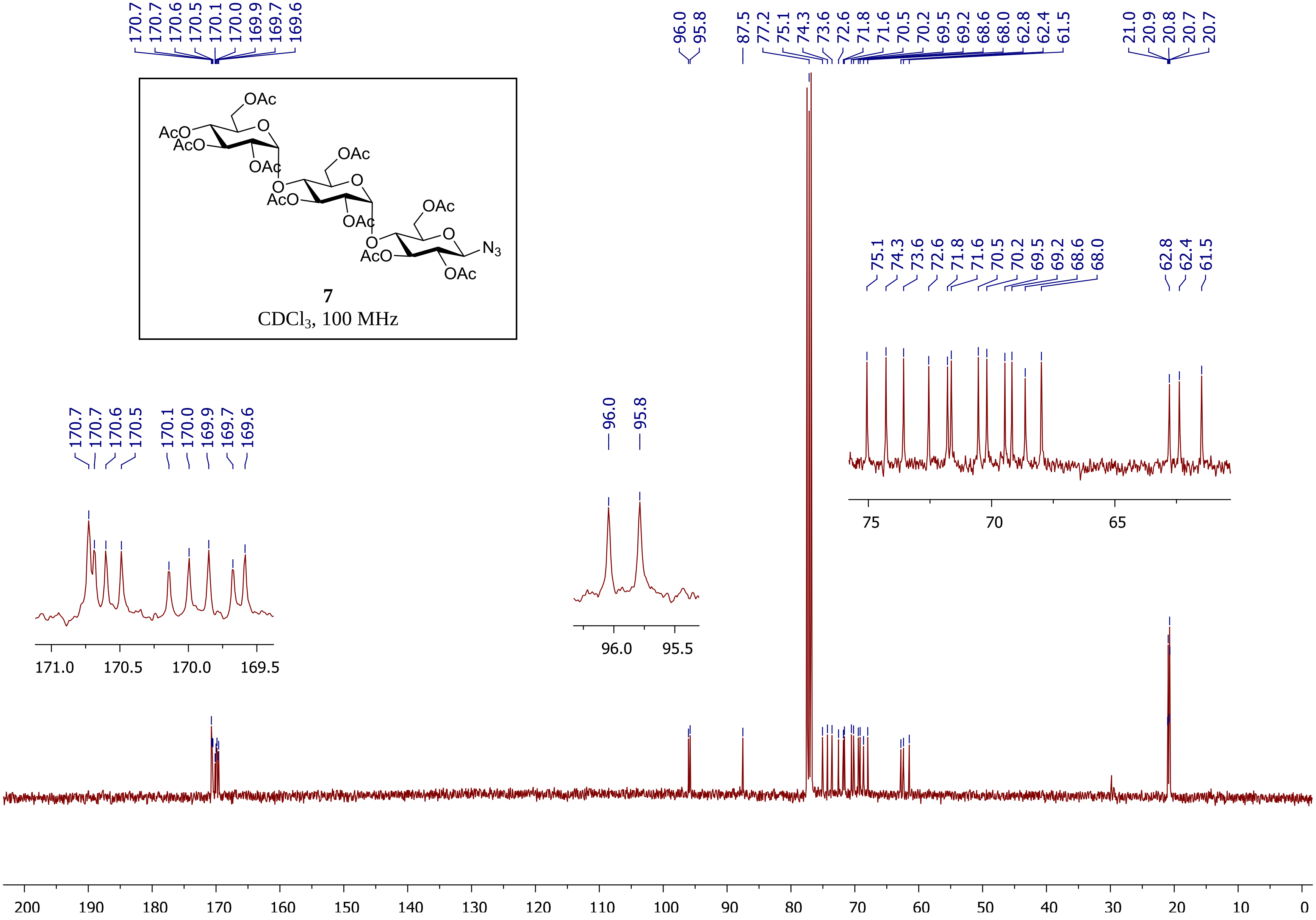
170.7 170.7 170.6 170.5 170.1 170.0 169.9 169.7 169.6
96.0 95.8
87.5 77.2 75.1 74.3 73.6 72.6 71.8 71.6 70.5 70.2 69.5 69.2 68.6 68.0 62.8 62.4 61.5
21.0 20.9 20.8 20.7 20.7
OAc
AcO
AcO
OAc
O
OAc
O
AcO
OAc
OAc
O
AcO
N3
OAc
7
CDCl3, 100 MHz
75.1 74.3 73.6 72.6 71.8 71.6 70.5 70.2 69.5 69.2 68.6 68.0
62.8 62.4 61.5
75 70 65
171.0 170.5 170.0 169.5
96.0 95.5
200 190 180 170 160 150 140 130 120 110 100 90 80 70 60 50 40 30 20 10 0

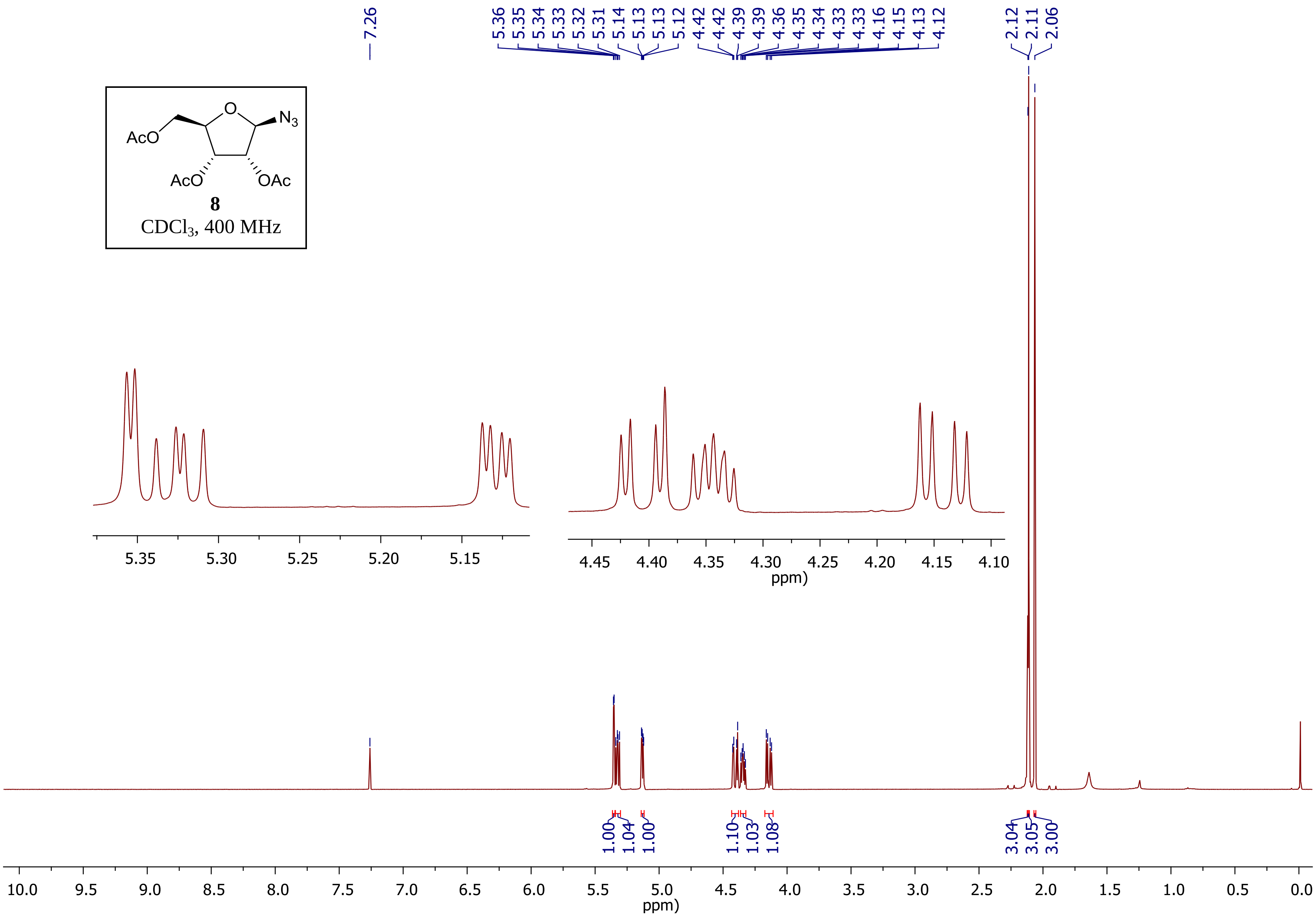
7.26
5.36 5.35 5.34 5.33 5.32 5.31 5.14 5.13 5.13 5.12 4.42 4.42 4.39 4.39 4.36 4.35 4.34 4.33 4.33 4.16 4.15 4.13 4.12
2.12 2.11 2.06
AcO
O
N3
AcO
OAc
8
CDCl3, 400 MHz
5.35 5.30 5.25 5.20 5.15
4.45 4.40 4.35 4.30 4.25 4.20 4.15 4.10
ppm)
1.00 1.04 1.00 1.10 1.03 1.08 3.04 3.05 3.00
10.0 9.5 9.0 8.5 8.0 7.5 7.0 6.5 6.0 5.5 5.0 4.5 4.0 3.5 3.0 2.5 2.0 1.5 1.0 0.5 0.0
ppm)

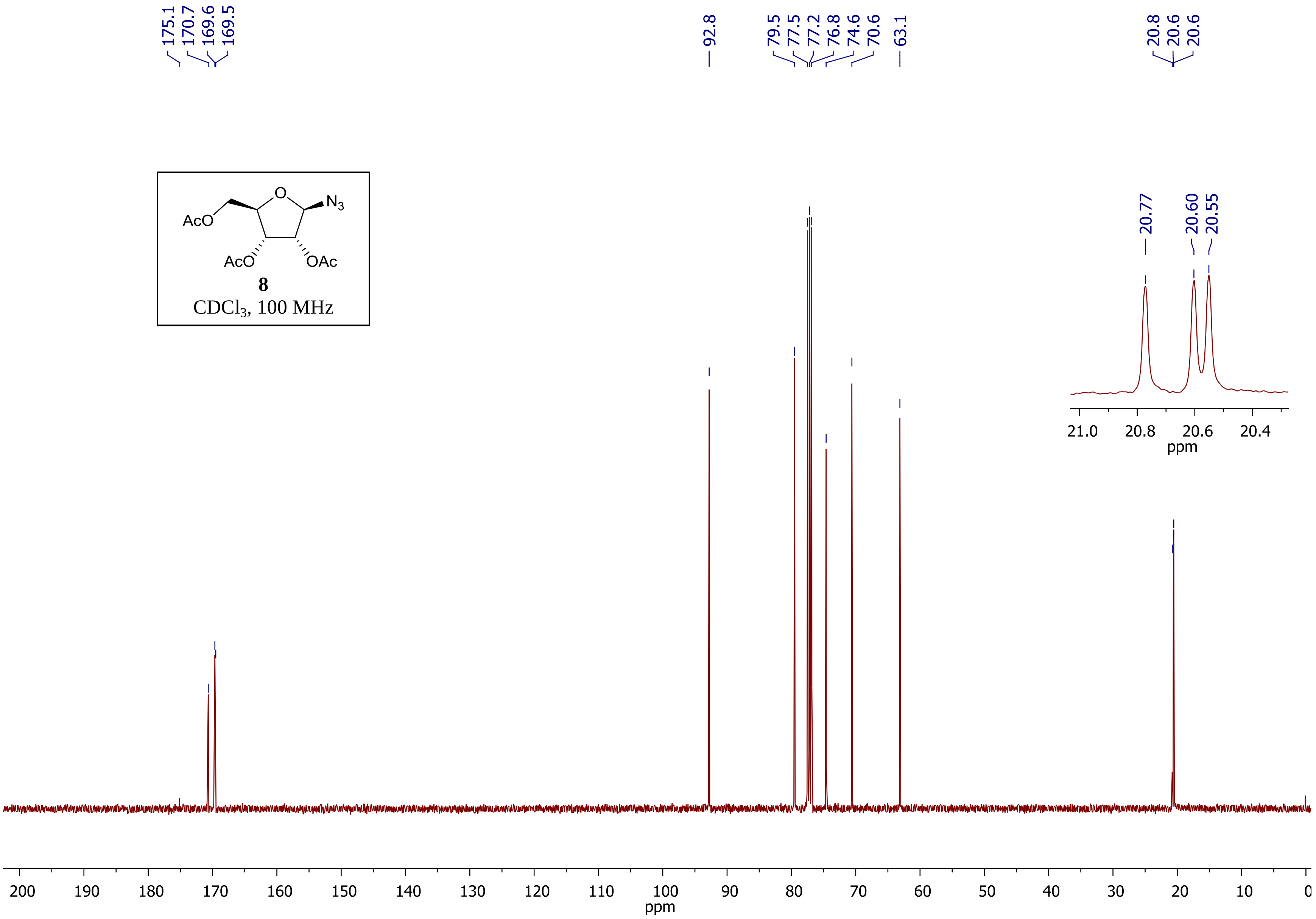
175.1
170.7
169.6
169.5
92.8
79.5
77.5
77.2
76.8
74.6
70.6
63.1
20.8
20.6
20.6
AcO
O
N3
AcO
OAc
8
CDCl3, 100 MHz
20.77
20.60
20.55
21.0
20.8
20.6
20.4
ppm
200
190
180
170
160
150
140
130
120
110
100
90
80
70
60
50
40
30
20
10
0
ppm

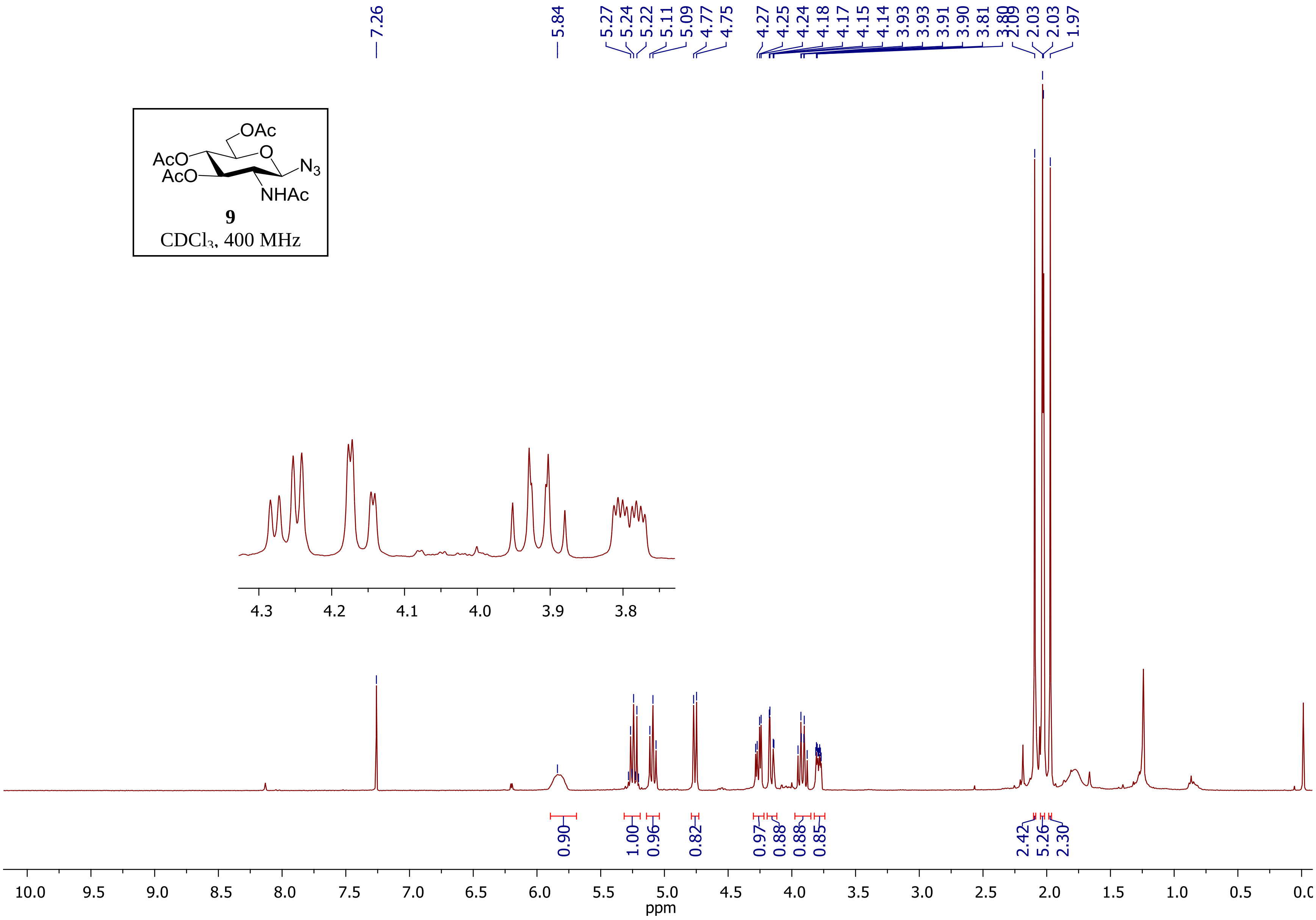
OAc
AcO
AcO
O
N3
NHAc
9
CDCl3, 400 MHz
ppm

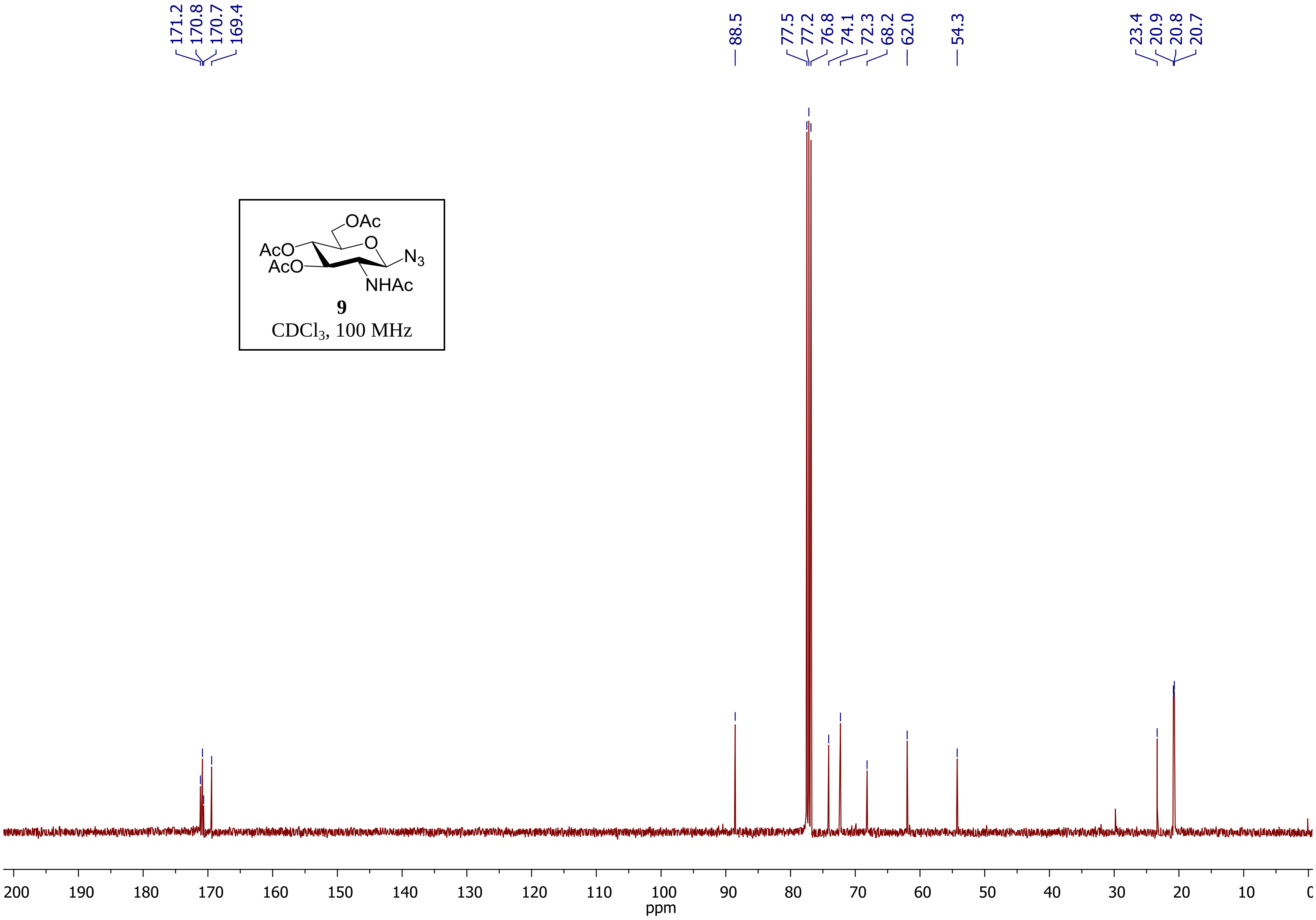
171.2
170.8
170.7
169.4
88.5
77.5
77.2
76.8
74.1
72.3
68.2
62.0
54.3
23.4
20.9
20.8
20.7
AcO
AcO
OAc
O
N_3
NHAc
9
$CDCl_3$, 100 MHz
200
190
180
170
160
150
140
130
120
110
100
90
80
70
60
50
40
30
20
10
ppm

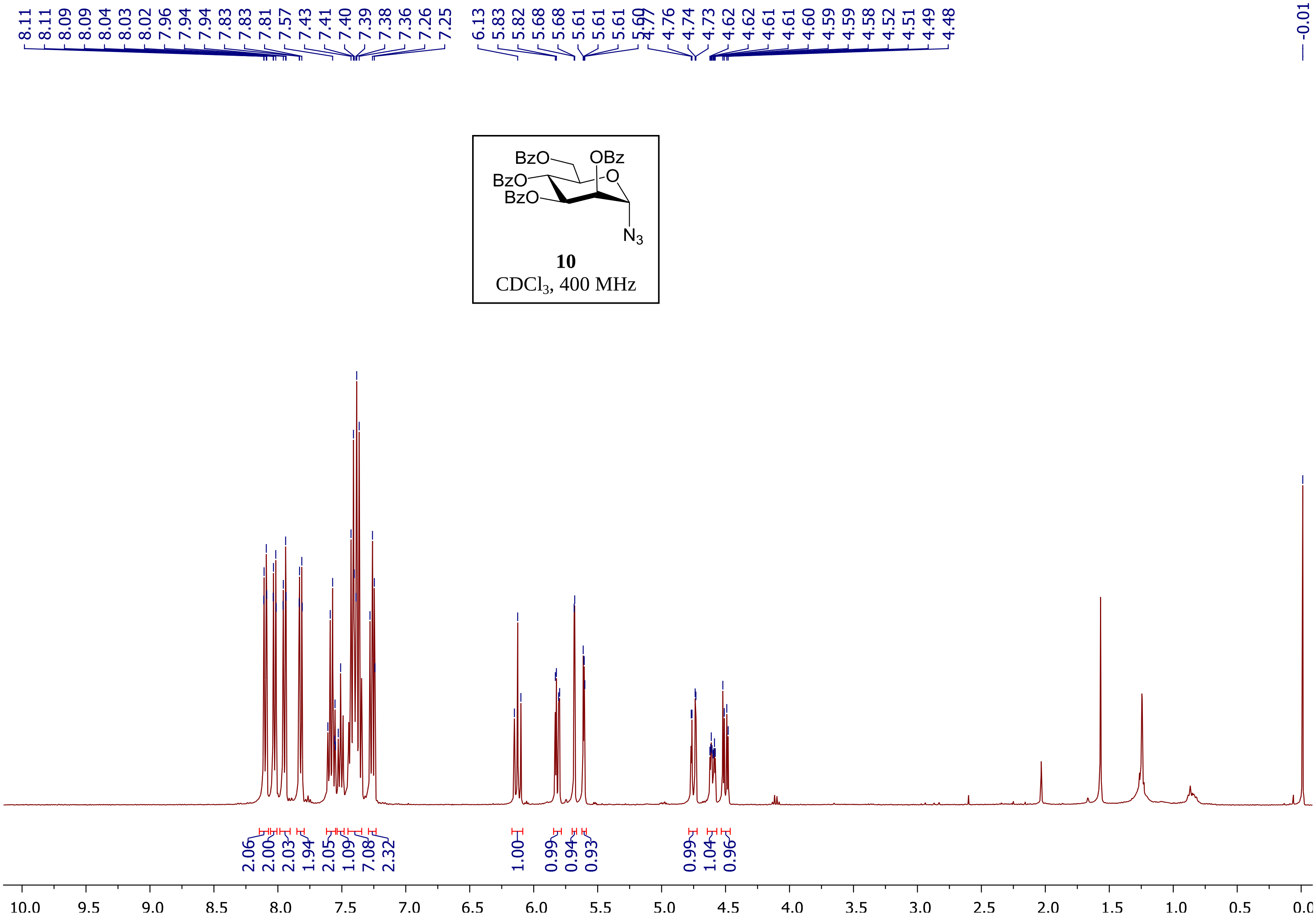
8.11 8.11 8.09 8.09 8.04 8.03 8.02 7.96 7.94 7.83 7.83 7.81 7.57 7.43 7.41 7.40 7.39 7.38 7.36 7.26 7.25
6.13 5.83 5.82 5.68 5.68 5.61 5.61 5.61 5.60 4.77 4.76 4.74 4.73 4.62 4.62 4.61 4.61 4.60 4.59 4.59 4.58 4.52 4.51 4.49 4.48
-0.01
BzO
OBz
BzO
BzO
O
N3
10
CDCl3, 400 MHz
2.06 2.00 2.03 1.94 2.05 1.09 7.08 2.32
1.00
0.99 0.94 0.93
0.99 1.04 0.96
10.0 9.5 9.0 8.5 8.0 7.5 7.0 6.5 6.0 5.5 5.0 4.5 4.0 3.5 3.0 2.5 2.0 1.5 1.0 0.5 0.0

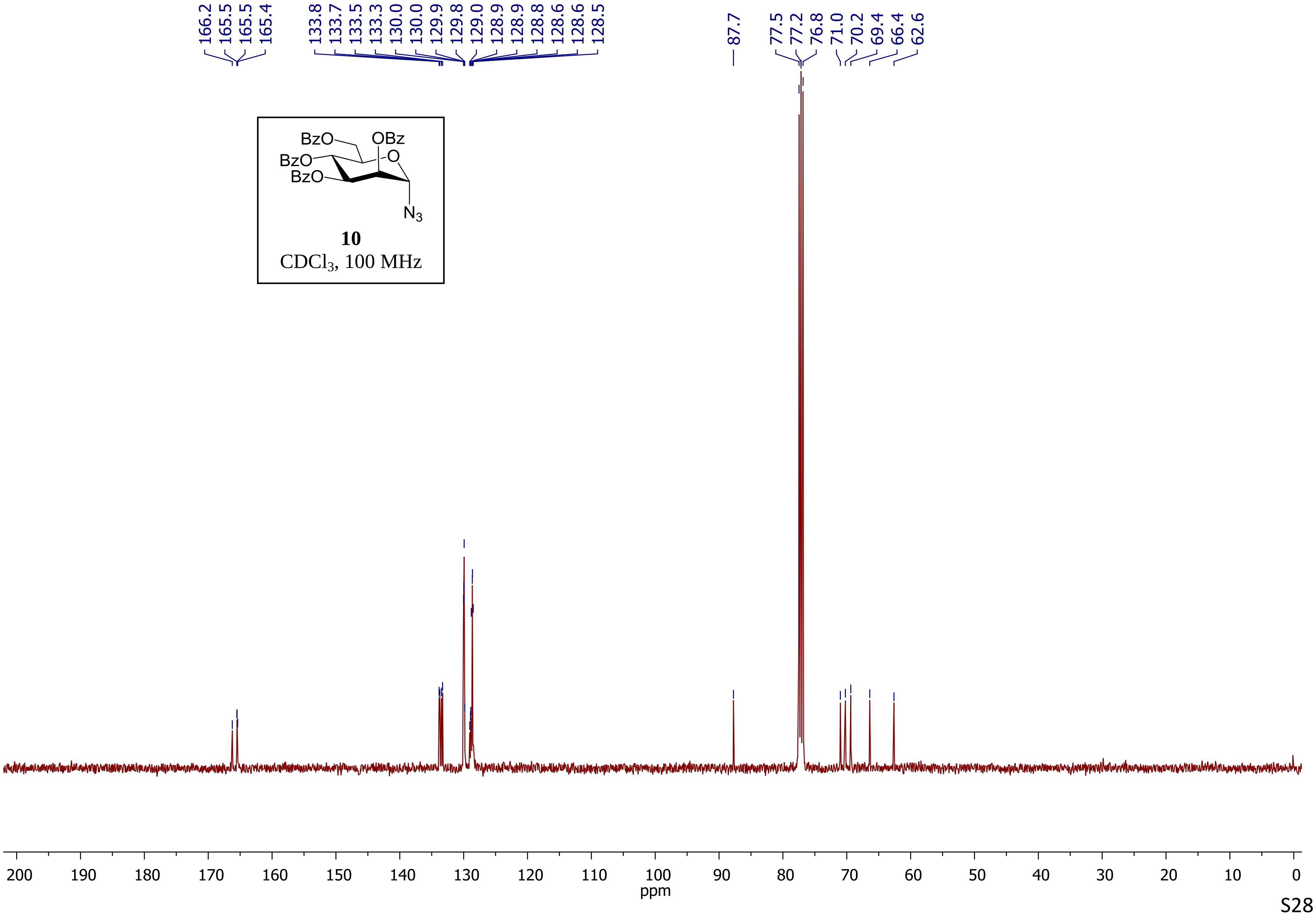
166.2
165.5
165.5
165.4
133.8
133.7
133.5
133.3
130.0
130.0
129.9
129.8
129.0
128.9
128.9
128.8
128.6
128.6
128.5
87.7
77.5
77.2
76.8
71.0
70.2
69.4
66.4
62.6
BzO
OBz
BzO
BzO
O
N3
10
CDCl3, 100 MHz
200 190 180 170 160 150 140 130 120 110 100 90 80 70 60 50 40 30 20 10 0
ppm

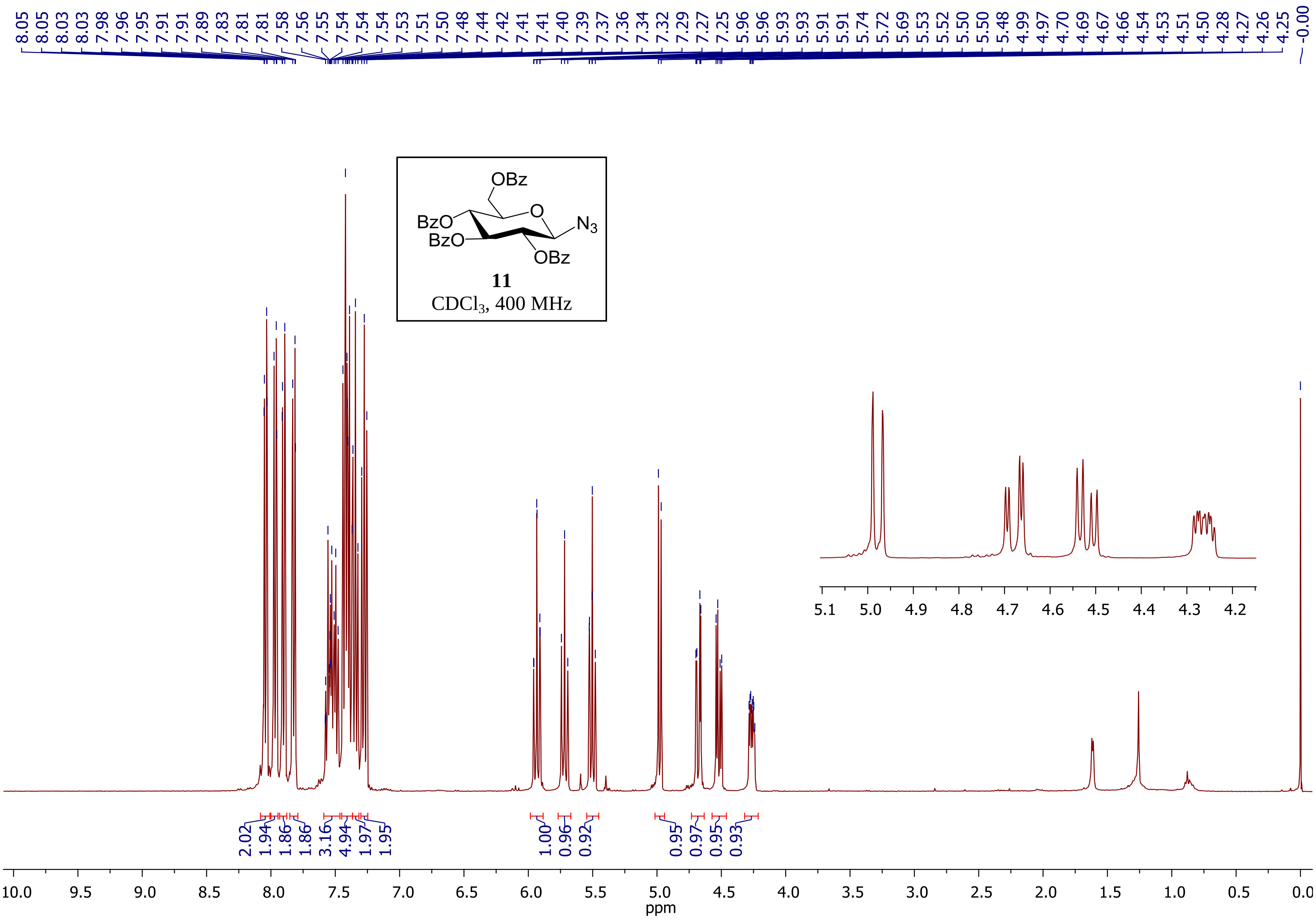
OBz
BzO
BzO
O
N_3
OBz
11
$CDCl_3$, 400 MHz
ppm

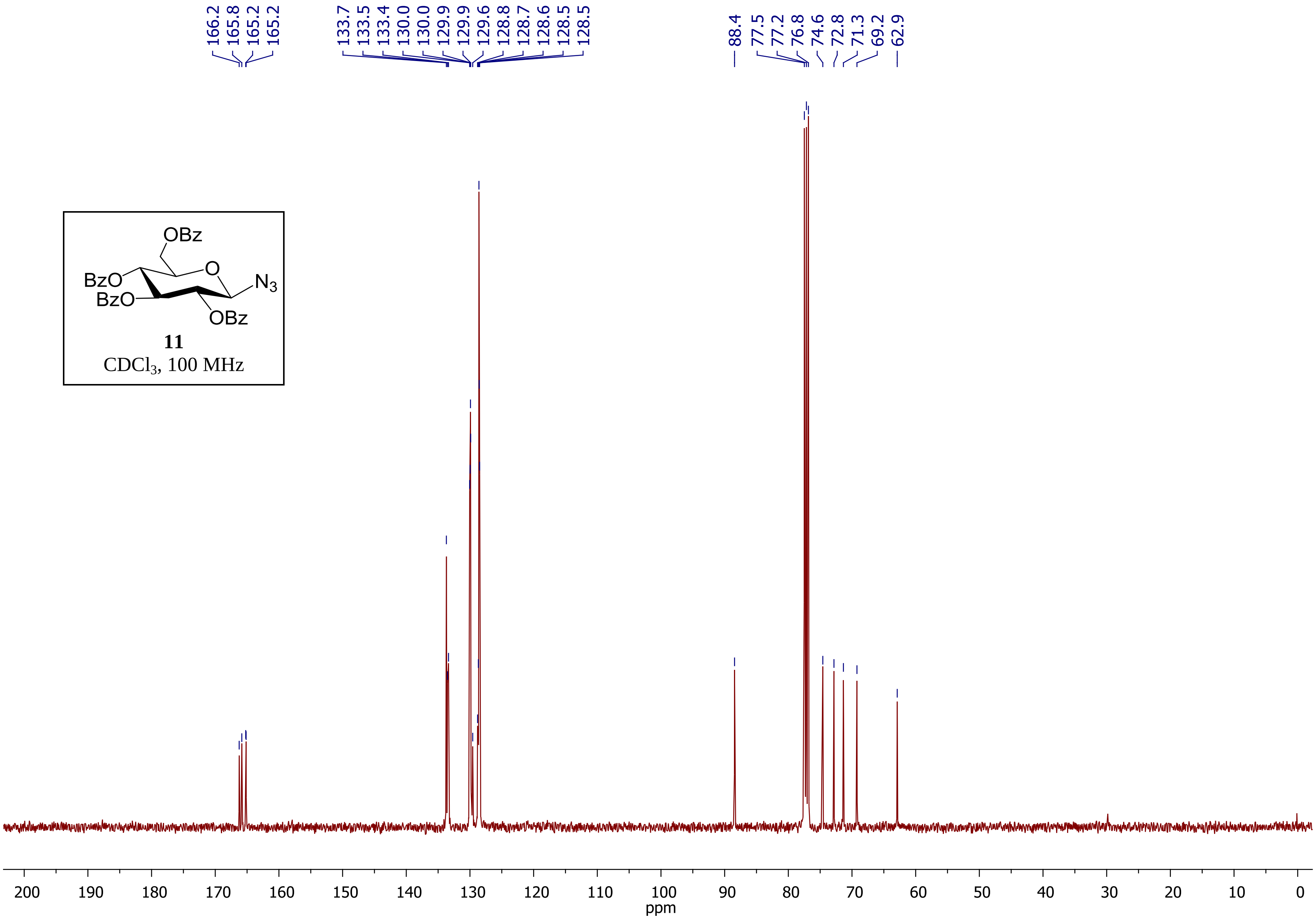

166.2
165.8
165.2
165.2
133.7
133.5
133.4
130.0
130.0
129.9
129.9
129.6
128.8
128.7
128.6
128.5
128.5
88.4
77.5
77.2
76.8
74.6
72.8
71.3
69.2
62.9
OBz
O
BzO
N3
BzO
OBz
11
CDCl3, 100 MHz
200
190
180
170
160
150
140
130
120
110
100
90
80
70
60
50
40
30
20
10
0
ppm

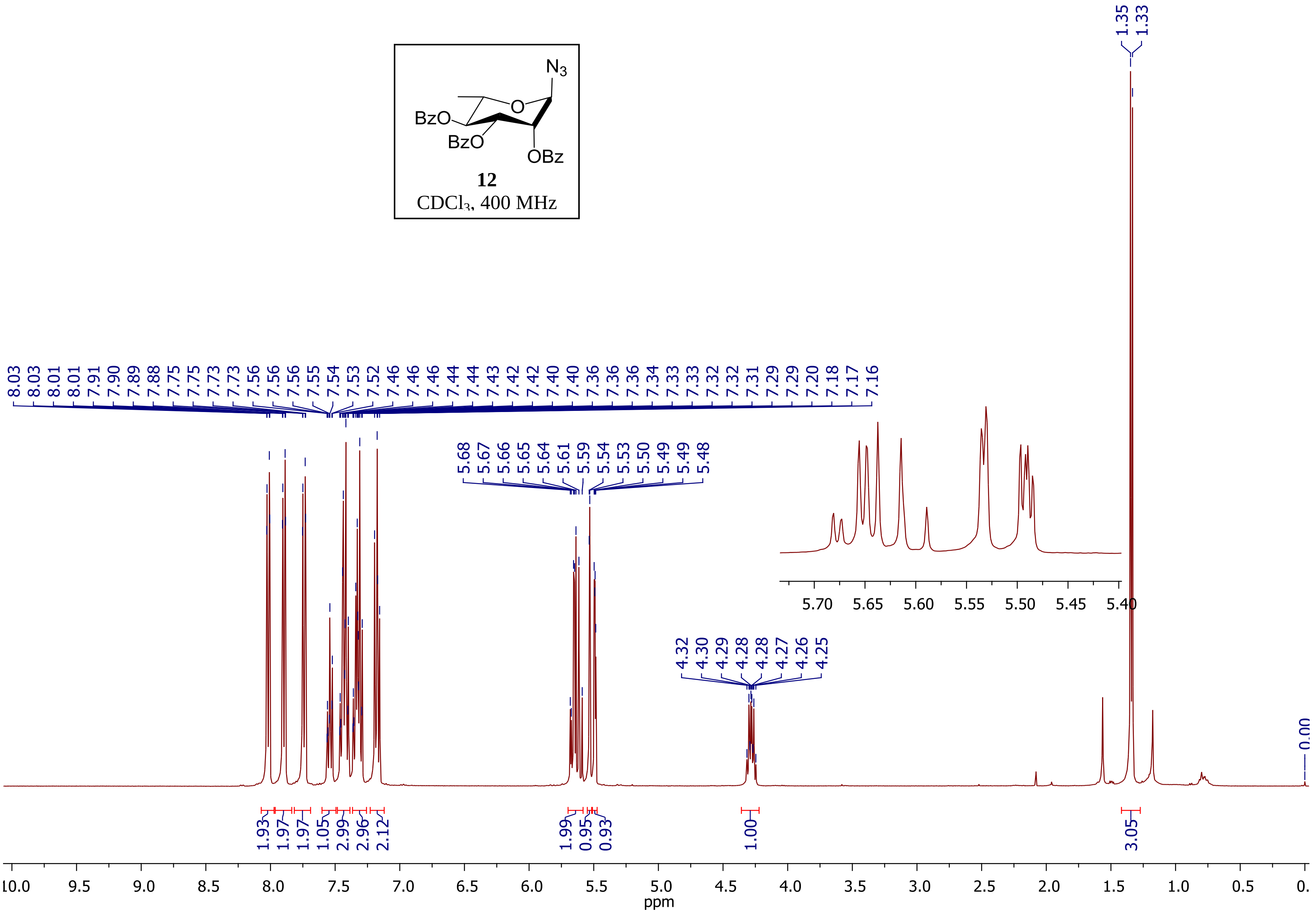
N3
BzO
BzO
O
OBz
12
CDCl3, 400 MHz
8.03 8.03 8.01 8.01 7.91 7.90 7.89 7.88 7.75 7.75 7.73 7.73 7.56 7.56 7.56 7.55 7.54 7.53 7.52 7.46 7.46 7.46 7.44 7.44 7.43 7.42 7.42 7.40 7.40 7.36 7.36 7.36 7.34 7.33 7.33 7.32 7.32 7.31 7.29 7.29 7.20 7.18 7.17 7.16
5.68 5.67 5.66 5.65 5.64 5.61 5.59 5.54 5.53 5.50 5.49 5.49 5.48
4.32 4.30 4.29 4.28 4.28 4.27 4.26 4.25
1.35 1.33
0.00
5.70 5.65 5.60 5.55 5.50 5.45 5.40
1.93 1.97 1.97 1.05 2.99 2.96 2.12 1.99 0.95 0.93 1.00 3.05
10.0 9.5 9.0 8.5 8.0 7.5 7.0 6.5 6.0 5.5 5.0 4.5 4.0 3.5 3.0 2.5 2.0 1.5 1.0 0.5 0.
ppm

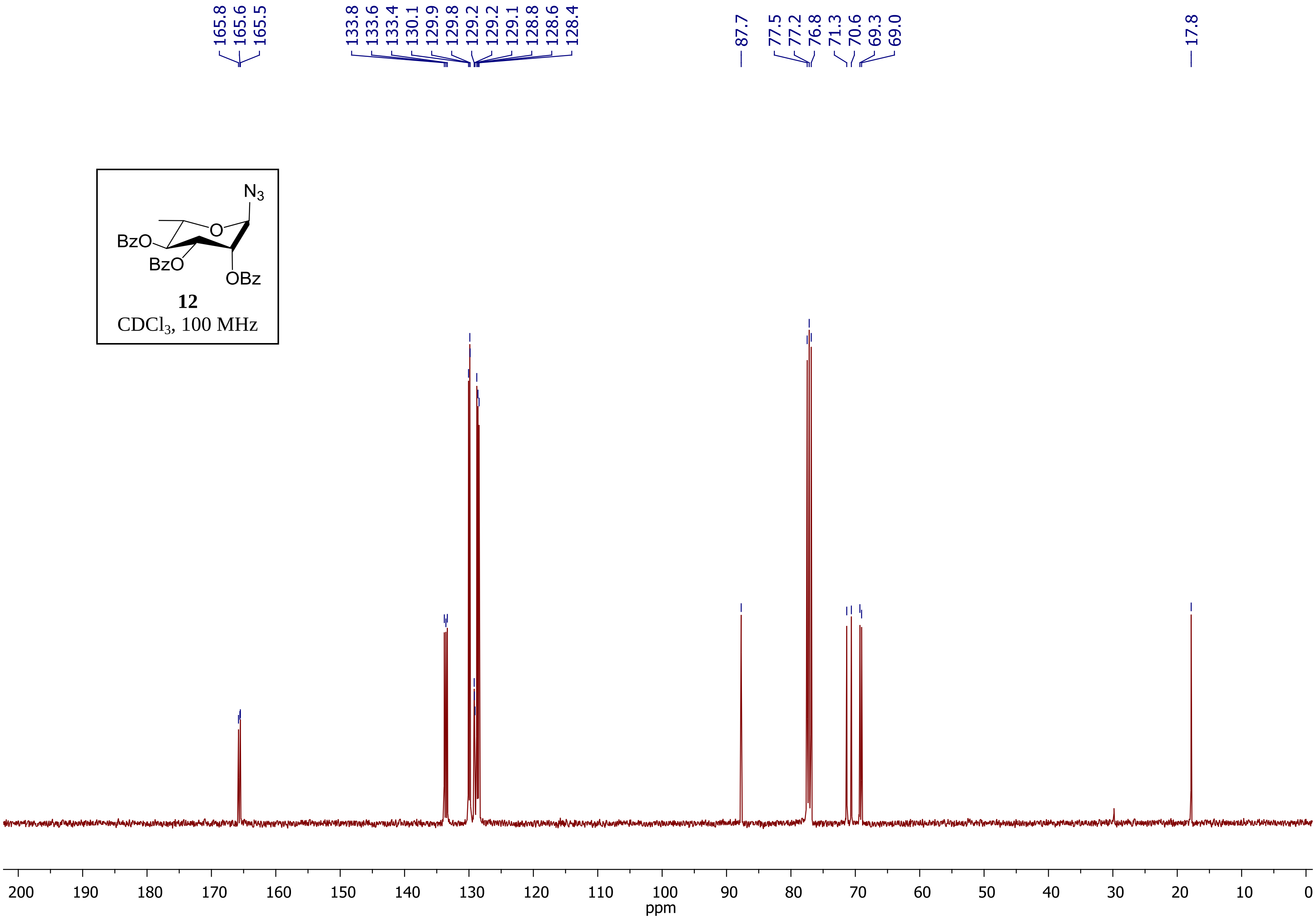
165.8
165.6
165.5
133.8
133.6
133.4
130.1
129.9
129.8
129.2
129.2
129.1
128.8
128.6
128.4
87.7
77.5
77.2
76.8
71.3
70.6
69.3
69.0
17.8
N3
BzO
BzO
O
OBz
12
CDCl3, 100 MHz
200
190
180
170
160
150
140
130
120
110
100
90
80
70
60
50
40
30
20
10
0
ppm

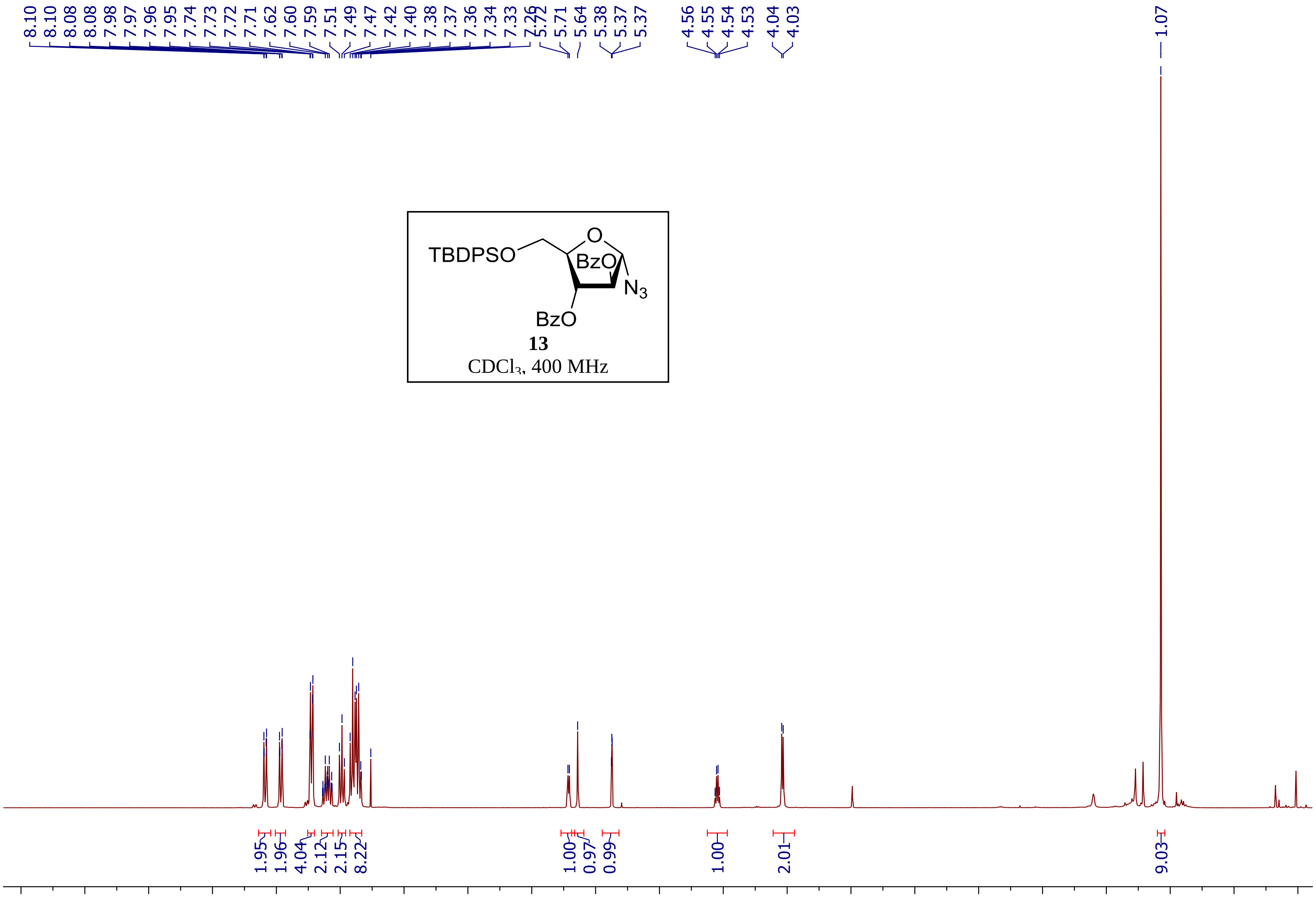
8.10 8.10 8.08 8.08 7.98 7.97 7.96 7.95 7.74 7.73 7.72 7.71 7.62 7.60 7.59 7.51 7.49 7.47 7.42 7.40 7.38 7.37 7.36 7.34 7.33 7.26 5.72 5.71 5.64 5.38 5.37 5.37 4.56 4.55 4.54 4.53 4.04 4.03 1.07
TBDPSO
BzO
N3
BzO
13
CDCl3, 400 MHz
1.95 1.96 4.04 2.12 2.15 8.22 1.00 0.97 0.99 1.00 2.01 9.03
10.0 9.5 9.0 8.5 8.0 7.5 7.0 6.5 6.0 5.5 5.0 4.5 4.0 3.5 3.0 2.5 2.0 1.5 1.0 0.5 0.0

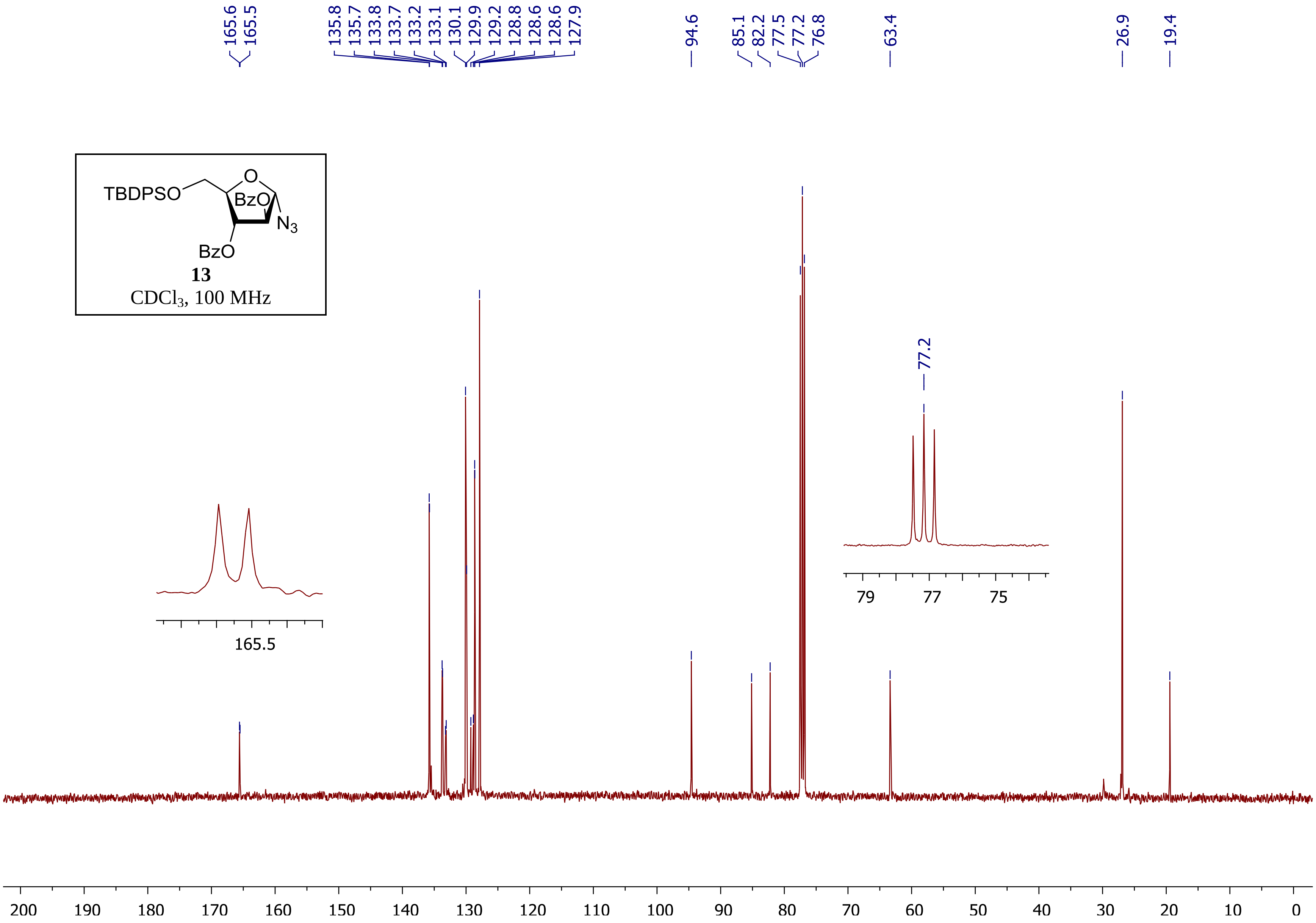
165.6
165.5
135.8
135.7
133.8
133.7
133.2
133.1
130.1
129.9
129.2
128.8
128.6
128.6
127.9
94.6
85.1
82.2
77.5
77.2
76.8
63.4
26.9
19.4
TBDPSO
O
BzO
N3
BzO
13
CDCl3, 100 MHz
165.5
77.2
79
77
75
200
190
180
170
160
150
140
130
120
110
100
90
80
70
60
50
40
30
20
10
0

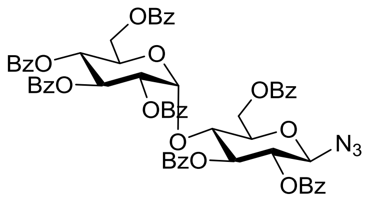

14

$CDCl_3$, 400 MHz

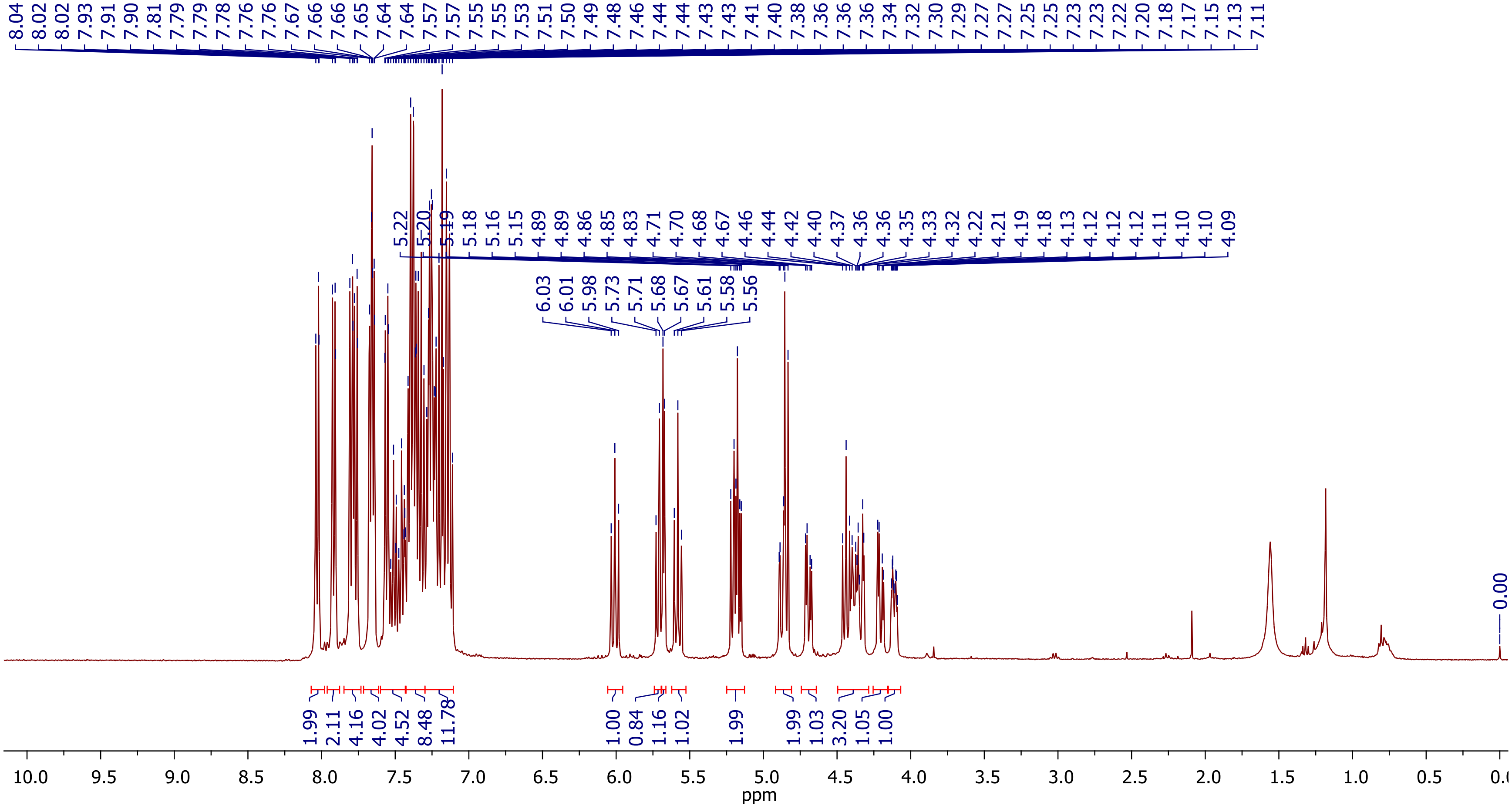

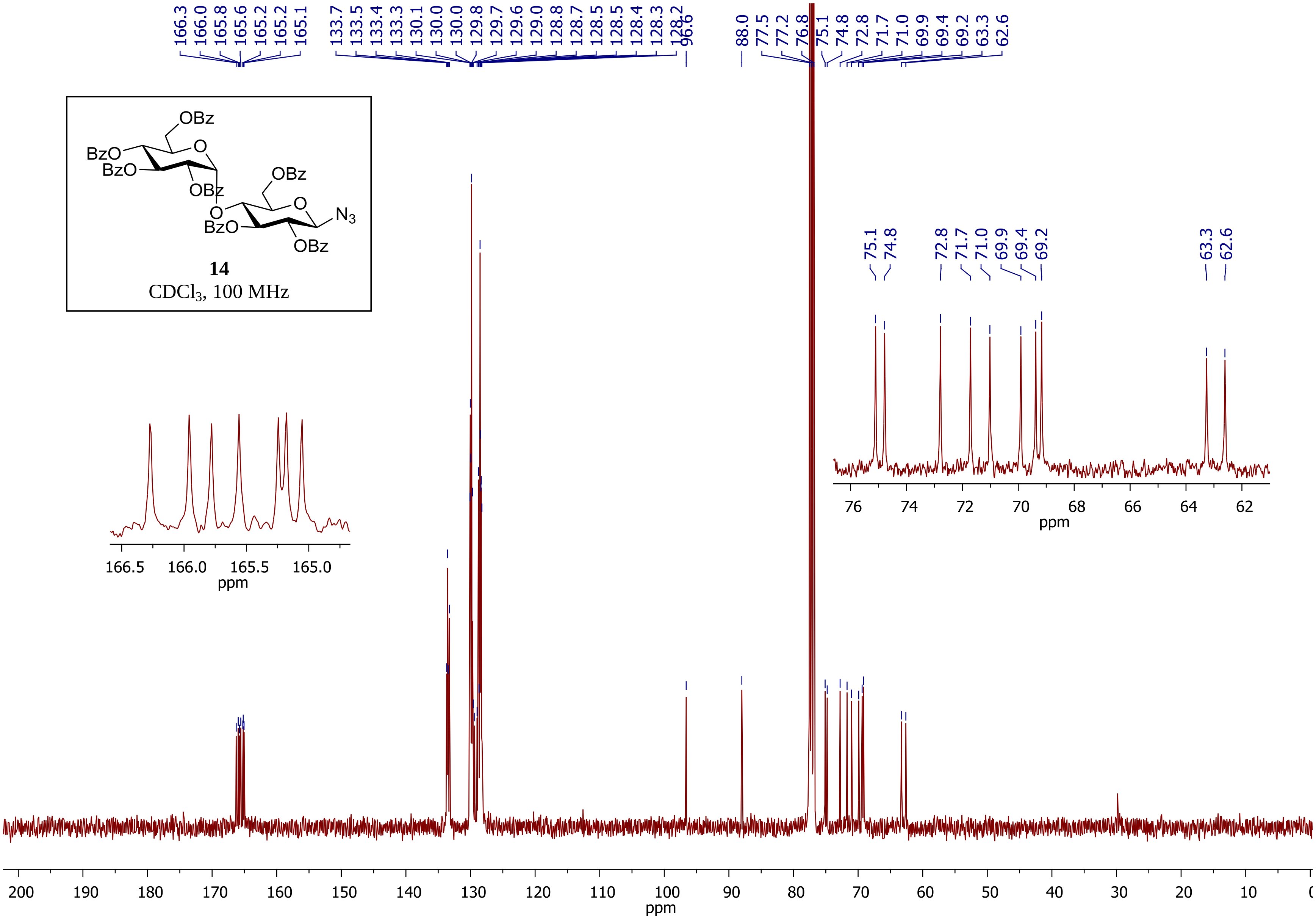
166.3
166.0
165.8
165.6
165.2
165.2
165.1
133.7
133.5
133.4
133.3
130.1
130.0
130.0
129.8
129.7
129.6
129.0
128.8
128.7
128.5
128.5
128.4
128.3
128.2
96.6
88.0
77.5
77.2
76.8
75.1
74.8
72.8
71.7
71.0
69.9
69.4
69.2
63.3
62.6
OBz
O
BzO
BzO
OBz
OBz
O
O
N3
BzO
OBz
14
CDCl3, 100 MHz
ppm
ppm
ppm

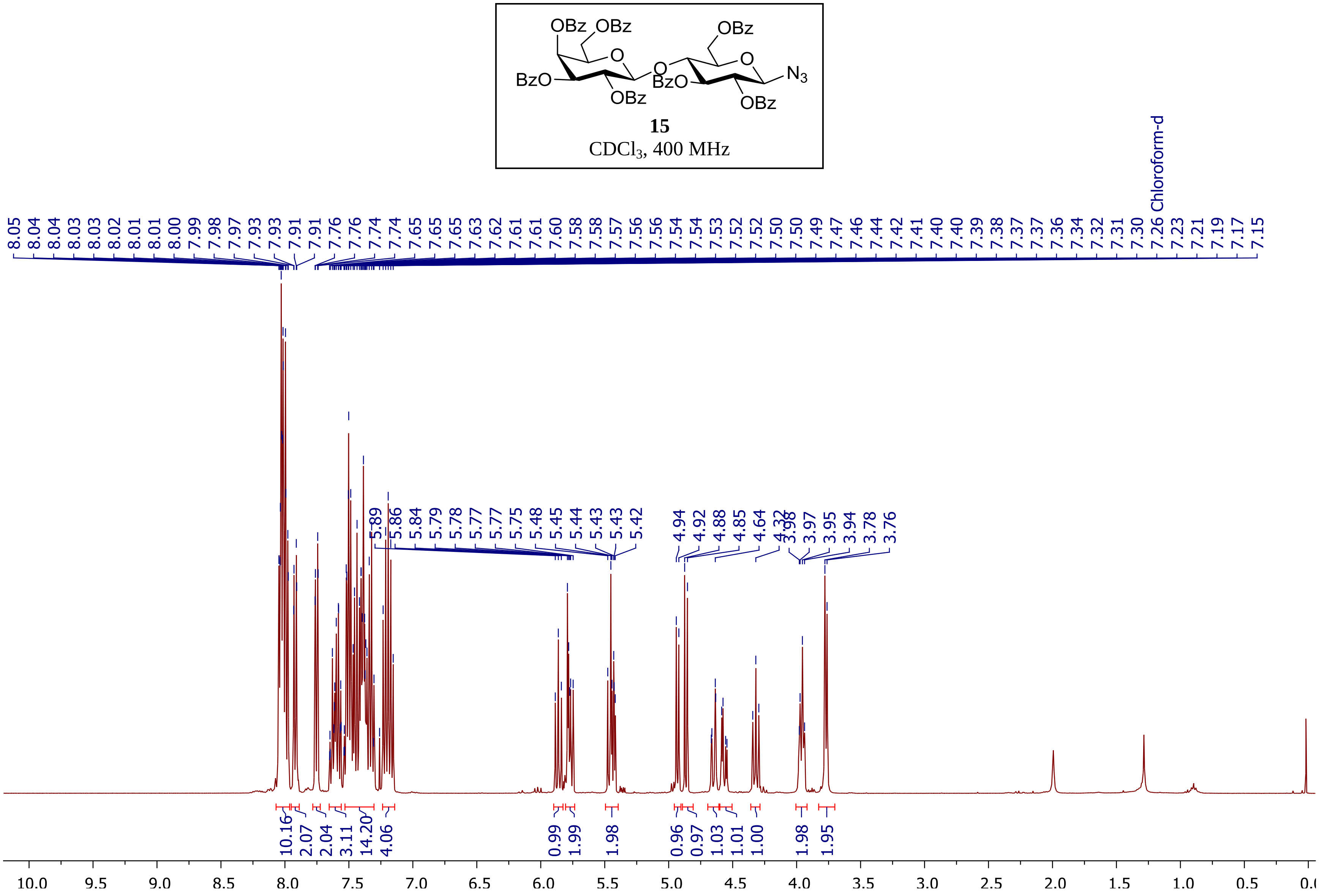
15
CDCl3, 400 MHz
Chloroform-d
10.16
2.07
2.04
3.11
14.20
4.06
0.99
1.99
1.98
0.96
0.97
1.03
1.01
1.00
1.98
1.95

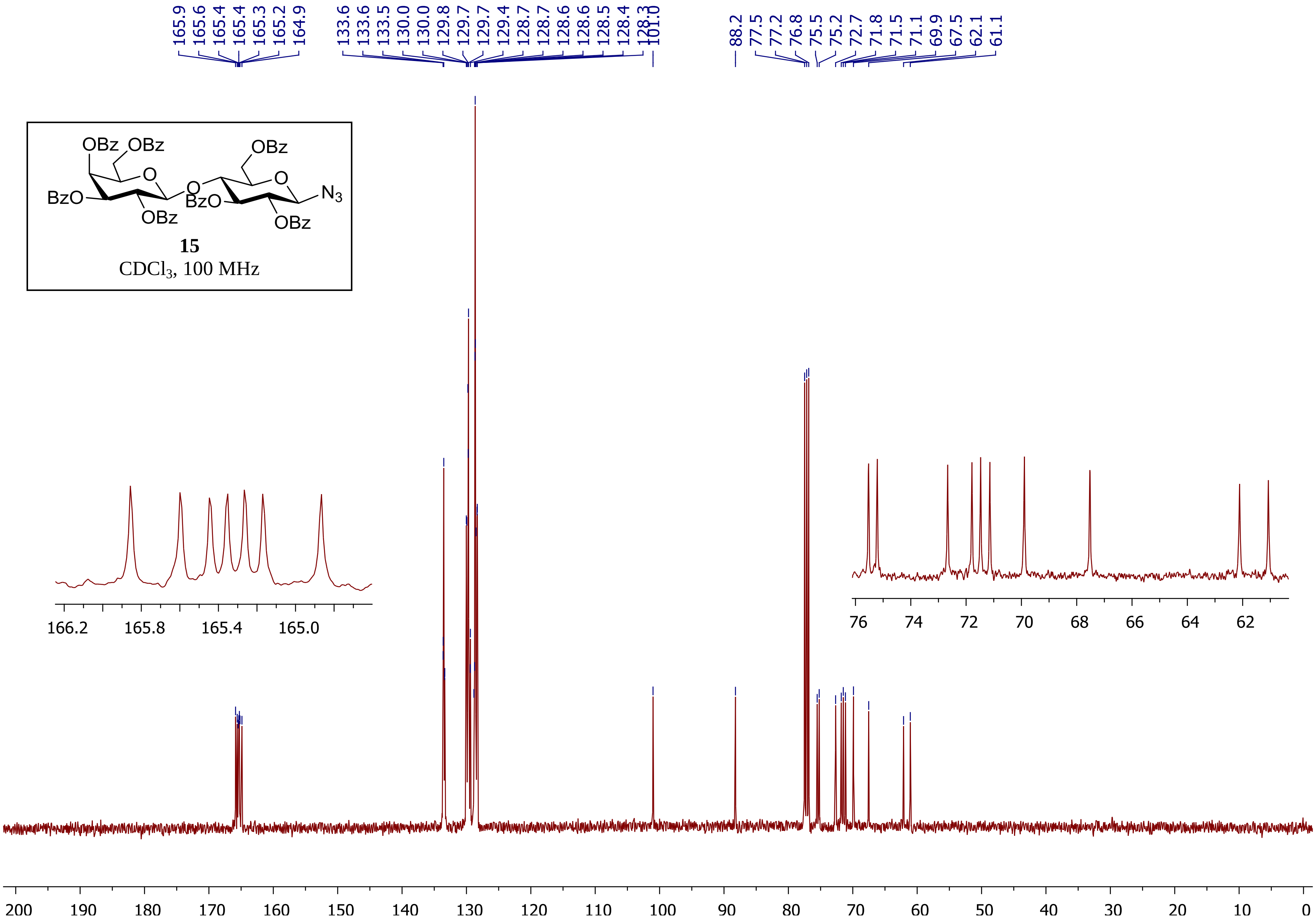

165.9 165.6 165.4 165.4 165.3 165.2 164.9
133.6 133.6 133.5 130.0 130.0 129.8 129.7 129.7 129.4 128.7 128.7 128.6 128.6 128.5 128.4 128.3 101.0
88.2 77.5 77.2 76.8 75.5 75.2 72.7 71.8 71.5 71.1 69.9 67.5 62.1 61.1
OBz
OBz
O
O
BzO
OBz
BzO
N3
15
CDCl3, 100 MHz
166.2 165.8 165.4 165.0
76 74 72 70 68 66 64 62
200 190 180 170 160 150 140 130 120 110 100 90 80 70 60 50 40 30 20 10 0
